# Modelling the Arrival of Invasive Organisms via the International Marine Shipping Network: A Khapra Beetle Study

**DOI:** 10.1371/journal.pone.0044589

**Published:** 2012-09-06

**Authors:** Dean R. Paini, Denys Yemshanov

**Affiliations:** 1 Cooperative Research Centre for National Plant Biosecurity, Bruce, Australia; 2 CSIRO, Ecosystem Sciences, Clunies Ross St, Acton, Australia; 3 Natural Resources Canada, Canadian Forest Service, Great Lakes Forestry Centre, Sault Ste. Marie, Ontario, Canada; Swansea University, United Kingdom

## Abstract

Species can sometimes spread significant distances beyond their natural dispersal ability by anthropogenic means. International shipping routes and the transport of shipping containers, in particular are a commonly recognised pathway for the introduction of invasive species. Species can gain access to a shipping container and remain inside, hidden and undetected for long periods. Currently, government biosecurity agencies charged with intercepting and removing these invasive species when they arrive to a county’s border only assess the most immediate point of loading in evaluating a shipping container’s risk profile. However, an invasive species could have infested a container previous to this point and travelled undetected before arriving at the border. To assess arrival risk for an invasive species requires analysing the international shipping network in order to identify the most likely source countries and the domestic ports of entry where the species is likely to arrive. We analysed an international shipping network and generated pathway simulations using a first-order Markov chain model to identify possible source ports and countries for the arrival of Khapra beetle (*Trogoderma granarium*) to Australia. We found Kaohsiung (Taiwan) and Busan (Republic of Korea) to be the most likely sources for Khapra beetle arrival, while the port of Melbourne was the most likely point of entry to Australia. Sensitivity analysis revealed significant stability in the rankings of foreign and Australian ports. This methodology provides a reliable modelling tool to identify and rank possible sources for an invasive species that could arrive at some time in the future. Such model outputs can be used by biosecurity agencies concerned with inspecting incoming shipping containers and wishing to optimise their inspection protocols.

## Introduction

Species can spread and establish in new regions at distances that far exceed the limit of an organism’s natural dispersal ability, but only if the species can find an appropriate vector to carry the organism beyond its biological spread range. This long-distance spread is often driven by human activities, usually by international trade and transportation [1], [2], which has increased dramatically in the last fifty years [2]. In particular, international marine shipping, which carries 90% of world trade [3], has been acknowledged as the primary means for the introduction of many invasive species [2], [4], [5].

International marine shipping networks therefore provide a prominent pathway for the introduction of invasive species and there are many cases to illustrate this. For example, the spread of Asian tiger mosquito, *Aedes albopictus* [6] and *Aedes japonicas* [7] to numerous countries are both believed to be caused by the international marine trade in used tires. The invasive pathogen, pine wilt disease is also believed to have been spread around the world via contaminated wood in ships [8]. Dutch elm disease arrived to the UK from Canada via the shipment of Rock Elm [9], and numerous marine species have been translocated around the world as a result of shipping [10].

Identification of the potential pathways and sources of human-assisted introductions of invasive organisms presents significant challenges because it often requires an understanding and quantification of the relevant socio-economic activities. Considering the immense amount of environmental and economic damage invasive species cause worldwide [11], [12], [13], it is important that researchers attempt to assess potential risks and identify likely origins of existing and future infestations. In this way, the government agencies responsible for protecting a country’s borders and natural resources from these invasions can use this information to prioritize surveillance and plan for post-detection mitigation efforts.

The analysis and modelling of trade and transportation networks is becoming an increasingly used method to assess the potential of organisms to establish at previously uninvaded areas [4], [5], [14], [15], [16]. Recent studies also link the analysis of complex trade and transportation networks with the application of simulation models of an organism’s spread and establishment [4], [5], [17], [18]. While an application of simulation models to estimate movement of invasive organisms is a common analytical approach [19], fewer studies have performed analyses and simulations in the domain of transportation or trade networks [4], [5], [17]. Note that network-based analyses could potentially provide more targeted assessments of long-distance human-mediated spread, which traditional spatial dispersal models do not predict well [20]–[23]. Furthermore, the potential of an invasive species being introduced at a location of interest (such as port of entry or urban area) can be estimated from each potential origin (such as a foreign port) and then all potential points of origin can be ranked to identify the ports and countries that are the most likely to be a source for a new incursion.

In this paper we analyse a complex network of international shipping routes using a first-order Markov chain model to identify potential origins of introduction (i.e. source countries and international foreign ports) for the highly invasive Khapra beetle (*Trogoderma granarium*) to Australia. We also identify and rank those Australian ports most likely to receive this invasive species. This analysis was undertaken with a probabilistic pathway model that describes the likelihood of the pest being moved from port *i* to port *j* as a linear function of the number of trips made by container ships through the segment *ij* and depicted as a matrix of the transmission probabilities *p_ij_*. This matrix is then used to simulate the sequential pathways of the invasive pest’s transmission from the ports of origin where the pest is known to occur to the locations of interest (i.e. Australian ports of entry).

The pathway modelling approach presented here has some similarities with the raster-based gravity models [24], [25], however our study uses directional, vector-based information and thus helps uncover the pathway “crossroads” and the ports - transit hubs through which the movement of infested cargoes is most likely. Because shipping containers are infrequently inspected during the transit, and may be transported over long distances before being opened [26], the use of marine traffic data helps better direct surveillance efforts to the most probable origin locations of accidental pest’s at the ports of entry.

The Khapra beetle is a pest of stored grain, causing significant economic damage across the world [27], and has been nominated as among 100 of the ‘World’s Worst’ invaders [28]. If the Khapra beetle became established in Australia, it could have a significant impact on the Australian grain industry via reduced yields and increased treatment costs [29].

As such, the Khapra beetle has been identified as a high priority exotic pest of the Australian grains industry by Plant Health Australia who have developed an industry biosecurity plan should there be an incursion [27]. This study aims to identify those countries and overseas ports most likely to be the source of a potential incursion. This will aid in the development of more effective surveillance and inspection efforts in order to prevent this pest establishing in Australia.

## Materials and Methods

### Data

We obtained data from the Lloyd’s Maritime Intelligence Unit (LMIU) detailing every fully cellular container ship that arrived into Australian ports between January 1^st^, 2002 and December 31^st^, 2007. This data set contained the previous ten ports of call for these container ships before arriving into one of 30 Australian ports, documenting 25,507 arrivals and departures of 557 container ships, with many ships arriving multiple times during this period. These ships travelled from and to 553 foreign ports in 126 countries.

In order to build the network of potential pathways of Khapra beetle (*Trogoderma granarium*) introductions to Australian ports, we used the world wide distribution of Khapra beetle from the CABI Crop Protection Compendium [30]. This distribution was used to identify the ports within the known species range. While the pest is found in 36 countries, the container ships arriving into Australian ports during this period only travelled to 24 of them (87 ports).

### Pathway Model

We estimated the potential of Khapra beetle to arrive with marine container vessels via the application of a pathway-based, first-order Markov model of Khapra beetle spread with marine container vessels through an international marine shipping network.

For this study, we assumed that some transmission potential existed between all intermediate locations within the shipping route. Consider a vessel route A–B–C–D, where A, B, and C denote the foreign ports of call and D is a destination port in Australia. Given that the ship has taken and unloaded containerized cargoes at each port, it is feasible to assume that the pest could be moved through the segments A–B, B–C, C–D, as well as segments A–C, B–D and A–D. Hence, we have decomposed all routes that included more than two nodes in to the combinations of unique segments *i* to *j*. This information then was used to assemble a database of unique pairs of “origin”-“destination” ports, *ij* and the associated numbers of vessels travelled through each particular segment *i* to *j*.

The records did not detail the actual number of containers that have been unloaded or loaded at each particular port; hence we assumed that each vessel would have similar capacity to carry the pest in a containerized cargo. We acknowledge that more detailed information on the cargo types in cellular containers and the tonnages of loaded/unloaded containers could improve the accuracy of the pathway predictions however this information was not available. No future forecast was imposed on the container shipment data; therefore, the analysis is best interpreted as showing the present-day entry potential of Khapra beetle.

In summary, each unique pathway segment, *i* to *j* had an associated number of trips between two given ports, *i* and *j*, *m_ij_*. We then rescaled the number of trips to the transmission rate value, *p_ij_*, of the beetle being moved from *i* to *j* over the six year survey period (2002–2007) based on the total number of container vessels travelling from *i* to *j*:
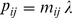
(1)where *λ_t_* is the rate of Khapra beetle transmission with one container vessel over the survey period *t* (six years). Essentially, *λ_t_* is a scaling coefficient that translates the number of trips to a transmission rate value so the sum of the transmission rates is below 1:



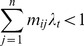
(2)The transmission matrix, **P** *_t_*, of the pest being moved along each pathway segment was then estimated accordingly:
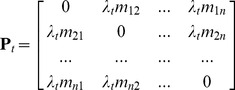
(3)


Note that the value of *λ_t_* is relative only to the number of vessels recorded in the Lloyds data between 2002 and 2007. Because the pathway model was only used in a relative context – to order the foreign ports by their *relative* potential to be the source of Khapra beetle incursions at Australian ports (i.e. that the port X has a higher potential to be the source of beetle incursions than the port Y) – a precise estimation of *λ_t_* was unnecessary. In our case, we selected the *λ_t_* value to ensure any row in the matrix P*_t_* satisfies the condition in Eq. 2 (so the row sum of the transmission rates is below 1). Note that the use of small *λ_t_* values that ensure the conditions Σ*p_ij_* <1 was done for technical reasons so the *λ_t_m_ij_* values could be treated as probabilities in the model simulations.

The matrix had a size, *Y*, equal to the number of port locations in the Lloyds register data (i.e. the nodes of the shipping network). The data did not provide information about the number of containers unloaded/loaded at intermediate ports, so the diagonal elements of **P** *_t_* were set to 0.

We also added an extra column to the matrix **P** *_t_* that describes the potential of the Khapra beetle not surviving transit from *i* to *j,* i.e.:
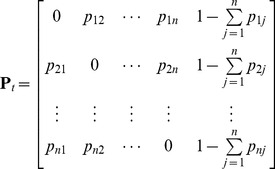
(4)where the elements 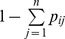 describe the probability that the Khapra beetle doesn't arrive in port *j.*

The pathway matrix **P**_t_ was then used to generate stochastic realizations of potential movements of the beetle from the foreign ports in regions where the Khapra beetle is known to exist. Starting at each port in the countries with Khapra beetle (one port at a time), the model simulated the subsequent movements of the beetle to other locations by extracting the associated vector of transmission rates from the matrix **P** *_t_* at each port’s location and using it to select the next port. The process continued until the chosen location had no outgoing paths recorded in the **P**_t_ or a terminal state was selected based on the elements 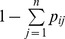
in Eq. 4. Finally, we estimated the rates of pest arrival from the location *i* from *j*, *φ_ij_*, from the number of times, *J_i_* the pathways originated at a given port *i* within a Khapra beetle range arrived at the port *j* over the multiple stochastic pathway simulations:
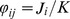
(5)where *K* is the total number of individual simulations of the pathway spread from *i* (*K* = 2×10^6^ for each port of origin *i*). Note that the value of *φ_ij_* was also estimated for each port in the network *i* outside of the Khapra beetle known range, however our study focuses on Australian ports only. Notably, the value of *φ_ij_* is conditional on the value of *λ* chosen. However, since *λ* was a linear multiplier applied to each element of the matrix **P** *_t_*, changes in its value did not affect the partial order relationships based on the estimated arrival rate values, *φ_ij_* and consecutively, the ranking of the individual ports by the Khapra beetle incursion potential.

### Summary Metrics

The forward-looking simulations provided for each port within the Khapra beetle range, *i*, list the arrival rates, *φ_ij_* to the destination ports *j* in Australia. For each port of origin *i* within the Khapra beetle range, we then compiled the lists of the arrival rate values, *φ_ij_* for all other “destination” locations in Australia, *j*, *j* = 1,…, *n*, *j* ≠ *i*. We then tabulated the *φ_ij_* values in a way so each “destination” location in the shipping network, *j* (i.e. an Australian port) had a corresponding list of the ports-potential origins, *i* (i.e. from where the pathway simulations were originally started) with the corresponding arrival rate values from the origin *i* to a destination *j*. We then summarized movement of the pest through the shipping network in two ways. First, for each Australian port, *j*, we generated an overall arrival rate of the Khapra beetle from all foreign ports as:
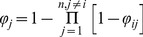
(6)where equation [6] is the product of all the beetle arrival rate values from all foreign ports to an Australian port, *j*.

We then used 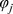 to rank the Australian ports by their potential to receive containers from the ports in the regions infested with Khapra beetle. We generated overall beetle arrival rates from each foreign port to all Australian ports combined. These values were then used to rank foreign ports by their potential to be the origin of a Khapra beetle infestation to Australian ports. It is tempting to identify arrival rates as arrival likelihoods, however these values are a function of λ, which currently is unknown. As such, we calculated relative arrival rate values, which give an indication of the relative difference in risk for foreign and Australian ports to receive the pest. These relative arrival rates were calculated by dividing a port’s arrival rate, *φ_ij_*, by the mean arrival rate.

We have also provided a general characteristic of the shipping network using the degree centrality metric. The degree centrality denotes the sum of the total numbers of ship arrivals and departures at a given port and is often interpreted as measure of the importance of a particular node in the network [31]. We then compared the degree centrality with the arrival rate, *φ_j_* for major Australian ports.

### Sensitivity Analysis

Uncertainties are an intrinsic feature of model-based assessments of ecological invasions [32], therefore it is important to estimate the impact of uncertainties on model results (the arrival rate values in our study). Uncertainty in the structure of the pathway network as well as key parameters of the pathway model can propagate in the model outputs [33], [34] and need to be properly estimated.

In this study we estimated the impact of the uncertainty in key elements of the pathway model on the port-specific arrival rates *φ_j_*. First, we tested two scenarios that considered somewhat different aspects of the uncertainty around the transmission rate values *p_ij_* in the pathway matrix **P**_t_ (Eq. 4). The first scenario added increasing random variation bounds around the *p_ij_* values but did not change the mean *p_ij_* values. Each pair of ± 0.3*p_ij_* bounds defined the endpoints for a symmetric uniform distribution (with an upper bound of *p_ij_* = 1.0 and lower bound of *p_ij_* = 0) from which we sampled values randomly for input into the model. This scenario explored the impact of multiplicative errors in *p_ij_* at the levels proportional to the baseline *p_ij_* values, so the higher *p_ij_* values had larger levels of uncertainty (and vice versa). The addition of multiplicative errors alters all positive values of *p_ij_* but does not change the nodes with *p_ij_* = 0. This implies that the multiplicative errors do not add or remove additional pathways with positive pest’s transmission potential and do not change the general structure of the shipping network. In general terms, these errors can be interpreted as uncertainty associated with the measurement of the transmission rates but holding the assumption that the general structure of the shipping network, the knowledge of ports with known beetle infestations and the average trade flow values are well known.

The second scenario estimated the impact of additive errors in the transmission probability values by adding a small uniform random variate to *p_ij_* regardless of their absolute values (i.e. including the nodes with *p_ij_* = 0) and observing the impact on the location-specific arrival rates, *φ_j_*. This scenario changes the mean values of *p_ij_* and also adds new nodes to the shipping network (by changing the *p_ij_* = 0 to a small positive random value and subsequently, altering the configuration of the shipping network). In short, this scenario adds the geographically uniform random variation to each network’s topology by assuming a very low probability of pest’s transmission through each possible network segment *ij* with a set of bounds [0; 0.05]. In broad terms, these additive errors in the *p_ij_* values depict an increasing lack of knowledge about the *p_ij_* (i.e. from where and to where the ships with potentially infested cargoes may be travelling) that shifts the *p_ij_* values towards a uniform random distribution and thus changes the patterns of commodity flows and a configuration of the shipping network (the latter aspect was also the reason of choosing a relatively low upper bound, 0.05, of uniform distributions of *p_ij_* values).

We have also tested the topological stability of the shipping network. Our third scenario explored the impact of uncertainty about the configuration of the transportation network, which is the presence or absence of a particular node in the network. This approach goes beyond the traditional sensitivity analysis [35], [36] and focuses on changes in the network’s connectivity [37]. To keep the analysis consistent with the abovementioned multiplicative and additive error scenarios we used a relatively straightforward technique and simulated added errors to knowledge about the network’s connectivity by temporarily removing a random portion of interlinked paths *ij* from the network and observed the corresponding changes in arrival rate values, *φ*_j_. At each pathway simulation event, the proportion of nodes (i.e. elements *i,j* in the pathway matrix **P**_t_) to be removed randomly was drawn from a uniform distribution [0; 0.3]. In broad terms, the errors associated with the presence or absence of a particular node depict the potential lack of information about the connectivity of the shipping network (for instance, the insufficient data about undocumented intermediate stops in foreign ports before ship arrives to the final destination port).

## Results

### Rankings of Foreign Countries as Potential Origins of KB Arrivals

**Figure 1:**
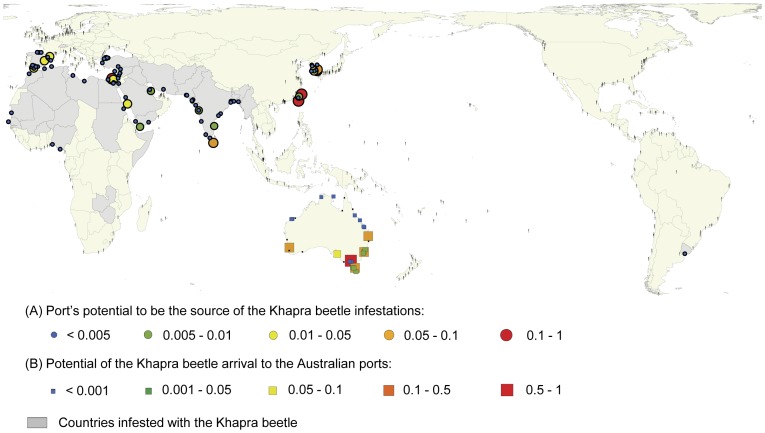
A geographical distribution of the Khapra beetle arrival potential to Australian ports. (A) Potential of foreign ports to be the source of Khapra beetle arrival at an Australian port, (B) The potential of Australian ports to receive Khapra beetle from foreign ports infested with the pest.

We found Taiwan and Republic of Korea had the highest potentials for being a source of Khapra beetle (*Trogoderma granarium*) incursions (Figure 1 and Table 1; for the full list see Table S1). The relative arrival rate from these two countries to Australian ports was considerably higher (more than 3 times) than the third highest-ranked country (Egypt).

**Table 1:** Top ten ranked source countries for Khapra beetle infestations at Australian ports.

Country	*φ_ij_*	Relative *φ_ij_* *
Taiwan	0.639	9.054
Republic of Korea	0.594	8.413
Egypt	0.155	2.197
Spain	0.096	1.355
Saudi Arabia	0.067	0.953
Sri Lanka	0.066	0.939
India	0.022	0.315
Yemen	0.013	0.186
Turkey	0.012	0.168
Pakistan	0.009	0.121

Countries ranked by the arrival rate (*φ_ij_*) to all Australian ports from the ports in a given country. For the full list see Table S1.

* denotes the pest’s relative arrival rate versus the avergae *φ_ij_* values for all network locations (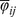 = 0.0706).

### Rankings of Australian Ports to Receive the Pest from Elsewhere

We found the port of Melbourne to have the greatest potential to receive containerized cargoes infested with Khapra beetle, with Botany Bay and Brisbane being the next two highest ranked ports, respectively (Figure 1 and Table 2; for the full list see Table S2).

We then examined the rankings of foreign ports for each of the ten Australian ports in Table 2 and found the ports of Busan (Republic of Korea) and Kaohsiung (Taiwan) to be ranked first and second (respectively) for nine of the ten Australian ports, with their order reversed at Botany Bay (Table 3; for full lists see Tables S3, S4, S5, S6, S7, S8, S9, S10, S11, S12). The relative rates of the Khapra beetle arrival to Australian ports from these two foreign ports are generally three or more times higher than from the third ranked foreign port.

**Table 2:** Top ten ranked Australian ports for receiving the Khapra beetle from foreign ports.

Australian Port	*φ_ij_*	Relative *φ_ij_* *
Melbourne	0.547	8.921
Botany Bay	0.398	6.487
Brisbane	0.390	6.369
Bell Bay	0.217	3.537
Fremantle	0.154	2.517
Adelaide	0.095	1.548
Burnie	0.050	0.808
Sydney	0.026	0.418
Hobart	0.014	0.225
Newcastle	0.003	0.047

Ports ranked by arrival rate of Khapra beetle (*φ_ ij_*) from foreign ports in the countries with known beetle presence. For the full list see Table S2.

* denotes the pest’s relative arrival rate versus the avergae *φ_ij_* values for all network locations (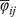 = 0.0613).

### Sensitivity Analysis

#### Multiplicative errors in the transmission probability values

**Figure 2:**
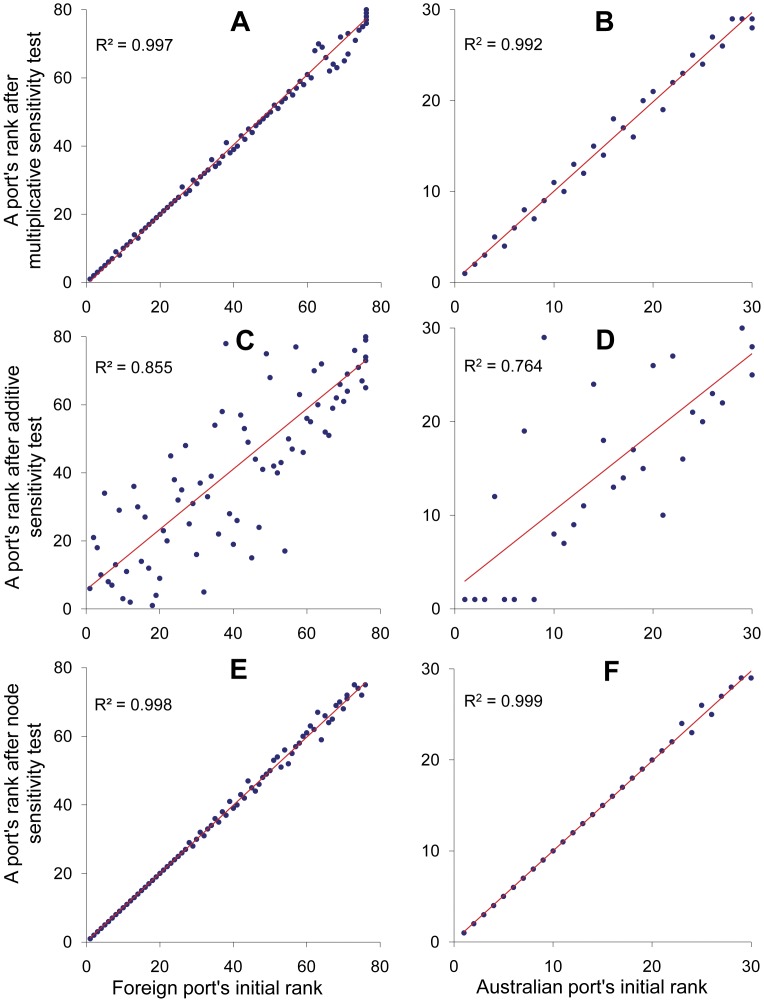
Sensitivity analysis. Changes in port rankings after the introduction of multiplicative errors (A–B), additive errors (C–D), and the random removal of a portion of the nodes from the transportation network (E–F). All figures show significant (p<0.001) rank correlations (see Results for details). The lowest rank values (starting from 1) indicate the highest risk.

We found the introduction of this type of error to the *p_ij_* values had little effect on the rankings of foreign ports, particularly those ports that were ranked in the top half of the list (Figure 2A: linear regression, t_85_ = 110.64, p<0.001, R^2^ = 0.997). A similar pattern was found with the ranking of Australian ports by their likelihoods to receive the Khapra beetle from foreign ports (Figure 2B: linear regression, t_29_ = 42.40, p<0.001, R^2^ = 0.992).

#### Additive errors in the transmission probability values

This introduced error changed the rankings of foreign and Australian ports (Figure 2C and 2D), though the rankings were still significantly correlated (foreign ports: linear regression, t_85_ = 15.19, p<0.001, R^2^ = 0.855; Australian ports: linear regression, t_29_ = 6.37, p<0.001, R^2^ = 0.764).

#### Random removal of nodes from the shipping network

The random removal of a proportion of nodes from the shipping network (and associated pairs of origin-destination ports) had little impact on the rankings. In particular, the rankings of the top 30 foreign ports remained unchanged (Figure 2E: linear regression, t_85_ = 155.22, p<0.001, R^2^ = 0.998). A similar pattern was observed for the rankings of Australian ports (linear regression, t_29_ = 114.23 at p<0.001, R^2^ = 0.999), with the ranks of the top 20 Australian ports remaining unchanged (Figure 2F).

## Discussion

### Major Threats of Khapra Beetle Arrivals

The two ports, Busan (Republic of Korea) and Kaohsiung (Taiwan) represent substantially greater threats for the arrival of Khapra beetle to Australia, than any other international port. Taiwan, was ranked as the greatest threat to Australia for this beetle, and has two of its ports (Kaohsiung and Keelung) ranked in the top three or four as potential sources of Khapra beetle arrivals to Australia (depending on the Australian port considered: Table 3). The high likelihood of ports in Taiwan and the Republic of Korea being a source of Khapra beetle is likely a result of a large number of container ships travelling through these two ports before arriving into Australia. Examining the port rankings by degree centrality (the total number of ship arrivals and departures at a given port) revealed that Busan and Kaoshing had approximately four times the degree centrality (3086 and 2979, respectively) of the third ranked port, Keelung (758). A similar pattern was observed when pooling degree centrality to the country level and examining the rankings. Again Taiwan and the Republic of Korea were the top two ranked by degree centrality.

**Table 3:** Port by port rankings.

Melbourne			Botany Bay			
Port of origin *i*	*φ_ij_*	Relative *φ_ij_* *	Port of origin *i*	*φ_ij_*	Relative *φ_ij_* *	
Busan (KOR)	0.245	94.601	Kaohsiung (TWN)	0.159	61.473	
Kaohsiung (TWN)	0.227	87.532	Busan (KOR)	0.158	61.020	
Keelung (TWN)	0.075	28.912	Keelung (TWN)	0.053	20.370	
Damietta (EGY)	0.039	14.925	Damietta (EGY)	0.025	9.702	
Colombo (LKA)	0.021	8.025	Colombo (LKA)	0.013	5.029	
Jeddah (SAU)	0.019	7.157	Jeddah (SAU)	0.012	4.704	
Valencia (ESP)	0.018	6.916	Valencia (ESP)	0.012	4.593	
Ulsan (KOR)	0.017	6.510	Port Said (EGY)	0.007	2.673	
Port Said (EGY)	0.011	4.160	Barcelona (ESP)	0.005	1.737	
Barcelona (ESP)	0.007	2.569	Ulsan (KOR)	0.004	1.500	
**Brisbane**			**Bell Bay**			
**Port of origin ** ***i***	***φ_ij_***	**Relative ** ***φ_ij_*** *	**Port of origin ** ***i***	***φ_ij_***	**Relative ** ***φ_ij_*** *	
Busan (KOR)	0.160	61.541	Busan (KOR)	0.084	32.346	
Kaohsiung (TWN)	0.158	61.098	Kaohsiung (TWN)	0.070	27.181	
Keelung (TWN)	0.052	20.153	Keelung (TWN)	0.023	8.947	
Damietta (EGY)	0.023	9.043	Damietta (EGY)	0.012	4.681	
Colombo (LKA)	0.012	4.453	Ulsan (KOR)	0.010	3.849	
Jeddah (SAU)	0.010	3.715	Colombo (LKA)	0.007	2.511	
Valencia (ESP)	0.009	3.388	Jeddah (SAU)	0.006	2.307	
Port Said (EGY)	0.005	2.084	Valencia (ESP)	0.006	2.234	
Gwangyang (KOR)	0.004	1.611	Port Said (EGY)	0.003	1.274	
Ulsan (KOR)	0.004	1.497	Barcelona (ESP)	0.002	0.803	
**Fremantle**			**Adelaide**			
**Port of origin ** ***i***	***φ_ij_***	**Relative ** ***φ_ij_*** *	**Port of origin ** ***i***	***φ_ij_***	**Relative ** ***φ_ij_*** *	
Busan (KOR)	0.051	19.612	Busan (KOR)	0.029	11.092	
Kaohsiung (TWN)	0.042	16.364	Kaoshiung (TWN)	0.024	9.246	
Damietta (EGY)	0.012	4.653	Damietta (EGY)	0.009	3.558	
Keelung (TWN)	0.012	4.614	Keelung (TWN)	0.007	2.678	
Valencia (ESP)	0.009	3.369	Colombo (LKA)	0.006	2.197	
Colombo (LKA)	0.008	2.917	Jeddah (SAU)	0.005	2.022	
Jeddah (SAU)	0.007	2.847	Valencia (ESP)	0.005	1.919	
Port Said (EGY)	0.004	1.623	Port Said (EGY)	0.003	1.270	
Barcelona (ESP)	0.003	1.014	Barcelona (ESP)	0.002	0.615	
Gwangyang (KOR)	0.002	0.814	Algeciras (ESP)	0.001	0.444	
**Burnie**			**Sydney**			
**Port of origin ** ***i***	***φ_ij_***	**Relative ** ***φ_ij_*** *	**Port of origin ** ***i***	***φ_ij_***		**Relative ** ***φ_ij_*** *
Busan (KOR)	0.0177	6.8258	Busan (KOR)	0.0084		3.2404
Kaoshiung (TWN)	0.0149	5.7627	Kaoshiung (TWN)	0.0077		2.9682
Keelung (TWN)	0.0049	1.8764	Keelung (TWN)	0.0025		0.9821
Damietta (EGY)	0.0026	0.9920	Damietta (EGY)	0.0018		0.6922
Ulsan (KOR)	0.0021	0.8098	Colombo (LKA)	0.0009		0.3436
Colombo (LKA)	0.0014	0.5356	Jeddah (SAU)	0.0007		0.2680
Jeddah (SAU)	0.0012	0.4785	Valencia (ESP)	0.0007		0.2535
Valencia (ESP)	0.0012	0.4650	Port Said (EGY)	0.0005		0.1909
Port Said (EGY)	0.0007	0.2591	Ulsan	0.0004		0.1463
Barcelona (ESP)	0.0004	0.1726	Barcelona (ESP)	0.0003		0.1126
**Hobart**			**Newcastle**			
**Port of origin ** ***i***	***φ_ij_***	**Relative ** ***φ_ij_*** *	**Port of origin ** ***i***	***φ_ij_***		**Relative ** ***φ_ij_*** *
Busan (KOR)	0.0049	1.8767	Busan (KOR)	0.00089		0.34454
Kaoshiung (TWN)	0.0041	1.5825	Kaoshiung (TWN)	0.00082		0.31716
Keelung (TWN)	0.0014	0.5427	Keelung (TWN)	0.00028		0.10701
Damietta (EGY)	0.0007	0.2684	Damietta (EGY)	0.00018		0.06883
Ulsan (KOR)	0.0005	0.2113	Colombo (LKA)	0.00009		0.03605
Colombo (LKA)	0.0004	0.1521	Valencia (ESP)	0.00009		0.03335
Jeddah (SAU)	0.0004	0.1357	Jeddah (SAU)	0.00007		0.02564
Valencia (ESP)	0.0004	0.1350	Barcelona (ESP)	0.00005		0.01947
Port Said (EGY)	0.0002	0.0833	Port Said (EGY)	0.00004		0.01504
Barcelona (ESP)	0.0001	0.0488	Karachi (PAK)	0.00004		0.01407

Top ten ranked source ports for Khapra beetle introduction to the ten most threatened Australian ports (see the rankings of Australian ports in Table 2). For the full lists see Tables S3, S4, S5, S6, S7, S8, S9, S10, S11, S12.

* denotes the relative pest’s arrival rate versus the avergae *φ_ij_* values for all network locations (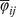 = 0.00259).

While the combined number of arrivals and departures (degree centrality) in this marine transportation network might be enough to identify ports (or countries) of high infestation risk, this may not always be sufficient to identify the gateways of pest introduction. The particular corridor of pest arrival will be a result of the configuration of the shipping routes (i.e. it becomes a function of a network’s topology). For example, the degree centrality could be distributed evenly within the network while some specific nodes (ports) may be part of a well-travelled transportation corridor that connects particular regions with considerable trade flows. In this case, undertaking the simulations of individual shipping pathways through the network (as we have done in this study) may be the only way to identify high ranked ports. This point can be illustrated by examining the lower ranked countries in Table 1. Yemen is ranked 8^th^, above Turkey and Pakistan, for arrival rate of Khapra beetle. However, using degree centrality, Yemen would be ranked below these two countries, with less than half the degree centrality (Yemen = 57 degree centrality, Pakistan = 125, Turkey = 113).

### Stability of the Ports’ Rankings to Uncertainty

The sensitivity analysis revealed that the rankings of Australian and foreign ports are considerably stable. Despite the uniform random variance introduced into the *p_ij_* values, the rankings remained relatively unchanged. These results are not surprising, given that the model is essentially a first-order Markovian pathway matrix, and the *p_ij_* values did not include geographically explicit or climate-specific modifications. Further research will be required to better determine how *p_ij_* (i.e. the transmission rate from one port to the next) might vary with season and geographical location. A better understanding of the value of *p_ij_* could mean these arrival rate values could be combined with establishment likelihoods [38] and serve as inputs into economic analyses and risk assessments associated with the particular groups of imports [39], [40], [41].

Indirect supportive evidence for this model comes from a Khapra beetle incursion in Perth, Western Australia in 2007. The beetle was found in the personal belongings of a family migrating to Australia from Scotland, which were shipped to Australia in an infected shipping container. A trace back of the container, which carried the family’s belongings revealed that the container had visited the following ports (in reverse order), Fremantle (Australia), Grangemouth (Scotland), Felixstowe (England), Pt Qasim (Pakistan), Gwangyang (South Korea), Busan (South Korea), Hamburg (Germany), Bangkok (Thailand) (personal communication – Rob Emery, Dept of Agriculture and Food, Western Australia, 2011). Although a molecular analysis to identify the exact source of this incursion was never completed, we note that Busan, and Gwangyang, were both visited by this container and were ranked 1^st^ and 10^th^ (respectively) as possible source ports for the receiving Australian port – Fremantle (Table 3).

### Potential Applications of the Pathway Model in Biosurveillance

Currently, Australian government biosecurity agencies do not identify a container’s previous ports (other than the immediate one in which the container was loaded before arriving to Australia). Considering the high risk posed by Khapra beetle and its ability to survive for long periods, undetected [42], biosecurity agencies should expand their screening efforts to collect a container’s pathway history from shipping companies and use it to evaluate a container’s risk profile. It would then be possible to optimise current inspection protocols and more efficiently allocate resources for biosecurity screening and surveillance.

Although the dataset used in our study documents the previous ten ports visited prior to the arrival of a ship to Australian ports, it is possible that the Khapra beetle could infest a container previous to these ten ports. The data show the average number of days at sea between ports is 7.9 days. Any ten-port steps would therefore cover, on average, 71 days. While this time frame would normally encompass the lifecycle of the Khapra beetle, its ability to go into a prolonged period of diapause could result in this species surviving long periods on a shipping journey [42], [43].

### Technical Aspects of an Application of the Pathway Model

The probabilistic pathway model presented in this study provides a computationally tractable and relatively simple way of incorporating trade and transportation data into assessments of human-assisted introductions of invasive pests. Essentially, the pathway model represents a network of vectors, each characterized by the probability of an organism’s transmission based on the frequency of container ships travelling between the ports in other countries and Australian ports of entry. The model uses directional marine traffic flows to estimate local rates of the invasive pest arrival at Australian ports. In general, the behavior of the model is similar to approximating the network of commodity marine traffic flows with a gravity model [5], however our model is focused on reconstructing the sequential pathways of movement of Khapra beetle with container ships and does not attempt to recreate the full topological structure of the marine shipping network.

The model presented here is a first-order Markov chain in which the next pathway segment taken by a container vessel is independent of its previous path. In short, when a container arrives into a port on a ship, its future path is not bound to that ship, but is considered a function of the number of ships travelling from that port to other ports. Clearly, this assumption could vary from port to port. For large hub ports, such as Singapore, where many arriving ships unload their containers for transfer to other ships, this is more likely to be a valid assumption. However, for smaller non-hub ports, most containers are likely to remain on a ship and continue along the predetermined path that ship travels. While presently, the vessel-specific data on container transfer were unavailable, we acknowledge that adding the port-specific estimates of the likelihood of a container being transferred to another ship after the arrival at the port would improve the pathway model and likely refine its predictions and rankings.

### Conclusions

The analysis of a marine shipping network represents a significant step forward in the assessment of pathways of entry and identification of potential source locations for invasive species, which previously only assessed direct pathways of entry from a source country. Considering the increasingly connected nature of the world’s transportation and trade networks, and the increasing multitude of potential carriers of invasive organisms from one part of the world to another, this more sophisticated evaluation of potential pathways would seem appropriate. Analysing trade and transportation networks using pathway model simulations enables the ranking of potential sources of invasive pest incursions, and can be applied to any invasive species of concern and any country at risk from invasion. Note that the method presented in this study does not attempt to find the source of infestations *per se* (as would be determined by genetic DNA testing methods), but rather prioritizes the most likely sources of future infestations from a multitude of potential locations of pest’s origins. Given the stability of the outputs of this model in the presence of uncertainty about the Khapra beetle’s port-to-port transmission potential, the analytic approach presented in this study helps improve and make more effective the current risk screening procedures of shipping containers undertaken by government agencies and industry stakeholders wishing to prevent the arrival and introduction of an invasive threat.

## Supporting Information

Table S1 **Ranking of source countries for Khapra beetle infestations at Australian ports.** Countries ranked by the arrival rate (*φ_ij_*) to all Australian ports from the ports in a given country.
(DOCX)

Table S2 **Ranking of Australian ports for receiving the Khapra beetle from foreign ports.** Ports ranked by arrival rate of Khapra beetle (φ ij) from foreign ports in the countries with known beetle presence.
(DOCX)

Table S3 **Ranking of all source ports for Khapra beetle introduction to the Australian port of Melbourne.**
(DOCX)

Table S4 **Ranking of all source ports for Khapra beetle introduction to the Australian port of Botany Bay.**
(DOCX)

Table S5 **Ranking of all source ports for Khapra beetle introduction to the Australian port of Brisbane.**
(DOCX)

Table S6 **Ranking of all source ports for Khapra beetle introduction to the Australian port of Bell Bay.**
(DOCX)

Table S7 **Ranking of all source ports for Khapra beetle introduction to the Australian port of Fremantle.**
(DOCX)

Table S8 **Ranking of all source ports for Khapra beetle introduction to the Australian port of Adelaide.**
(DOCX)

Table S9 **Ranking of all source ports for Khapra beetle introduction to the Australian port of Burnie.**
(DOCX)

Table S10 **Ranking of all source ports for Khapra beetle introduction to the Australian port of Sydney Harbour.**
(DOCX)

Table S11 **Ranking of all source ports for Khapra beetle introduction to the Australian port of Hobart.**
(DOCX)

Table S12 **Ranking of all source ports for Khapra beetle introduction to the Australian port of Newcastle.**
(DOCX)
