# Correction: Modelling the Arrival of Invasive Organisms via the International Marine Shipping Network: A Khapra Beetle Study

**DOI:** 10.1371/annotation/9f9b4966-1f98-492c-92bf-7e020ee4c006

**Published:** 2012-11-08

**Authors:** Dean R. Paini, Denys Yemshanov

There are formatting errors in Table 3. A correct Table 3 can be viewed here: 

**Figure:**
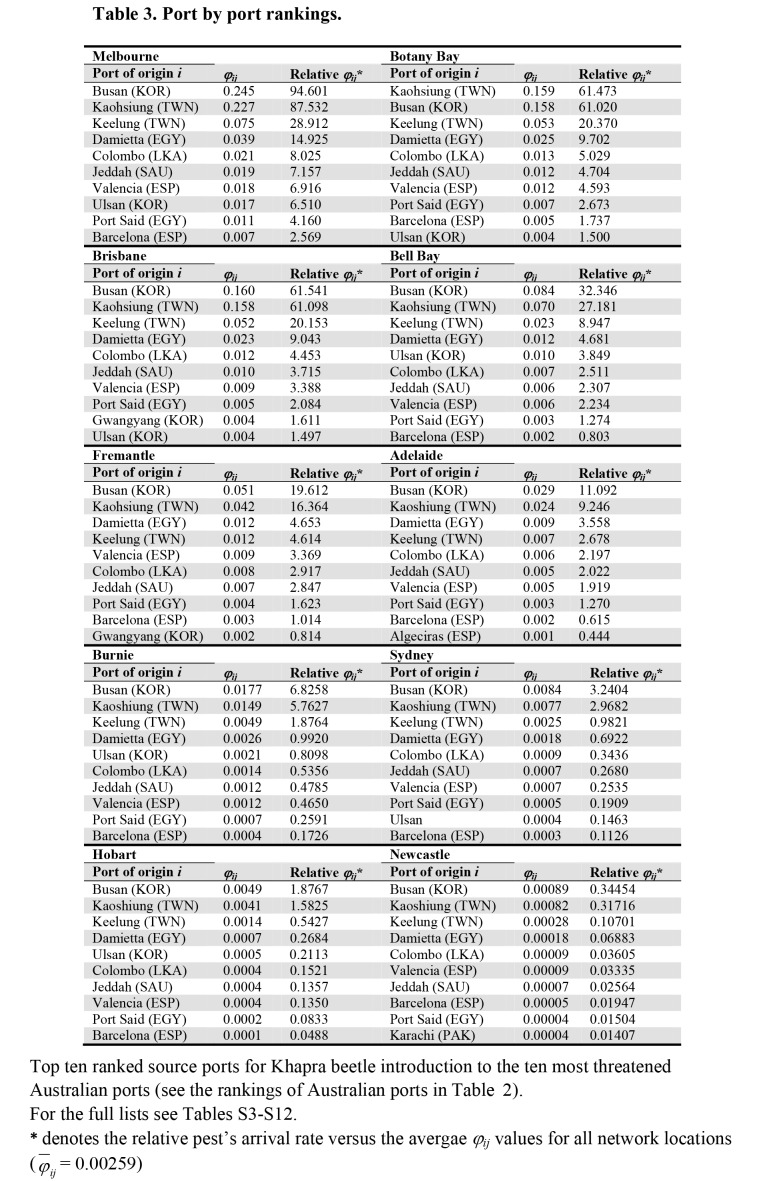


[^] 

