# Supplementary material for: Modelling the Arrival of Invasive Organisms via the International Marine Shipping Network: A Khapra Beetle Study
Source: PLoS One. 2012 Sep 6;7(9):e44589. doi: 10.1371/journal.pone.0044589 (PMC3435288; doi:10.1371/journal.pone.0044589)
Supplement: Table S1 — Ranking of source countries for Khapra beetle infestations at Australian ports. Countries ranked by the arrival rate (φij) to all Australian ports from the ports in a given country. (DOCX) [file pone.0044589.s001.docx]

Table S1. Ranking of source countries for Khapra beetle infestations at Australian ports. Countries ranked by the arrival rate (*ϕ_ij_*) to all Australian ports from the ports in a given country.

| **Country** | ***ϕ_ij_*** | **relative *ϕ_ij_**** |
| --- | --- | --- |
| Taiwan | 0.63928 | 9.05356 |
| Republic of Korea | 0.59408 | 8.41335 |
| Egypt | 0.15516 | 2.19732 |
| Spain | 0.09571 | 1.35546 |
| Saudi Arabia | 0.06730 | 0.95308 |
| Sri Lanka | 0.06634 | 0.93949 |
| India | 0.02222 | 0.31466 |
| Yemen | 0.01316 | 0.18635 |
| Turkey | 0.01184 | 0.16767 |
| Pakistan | 0.00852 | 0.12065 |
| Israel | 0.00576 | 0.08160 |
| Iran | 0.00419 | 0.05936 |
| Nigeria | 0.00308 | 0.04368 |
| Sudan | 0.00268 | 0.03791 |
| Cypress | 0.00229 | 0.03242 |
| Uruguay | 0.00099 | 0.01399 |
| Lebanon | 0.00075 | 0.01067 |
| Bangladesh | 0.00072 | 0.01018 |
| Algeria | 0.00032 | 0.00452 |
| Libya | 0.00026 | 0.00370 |
| Mauritania | 0.00003 | 0.00038 |
| Morocco | 0 | 0 |
| Senegal | 0 | 0 |
| Syria | 0 | 0 |
| **Mean** | **0.07061** |  |

***** denotes the relative pest’s arrival rate versus the avergae *ϕ_ij_* values for all network locations ($\overline{\phi}_{ij}$ = 0.07061)
