# Supplementary material for: Modelling the Arrival of Invasive Organisms via the International Marine Shipping Network: A Khapra Beetle Study
Source: PLoS One. 2012 Sep 6;7(9):e44589. doi: 10.1371/journal.pone.0044589 (PMC3435288; doi:10.1371/journal.pone.0044589)
Supplement: Table S2 — Ranking of Australian ports for receiving the Khapra beetle from foreign ports. Ports ranked by arrival rate of Khapra beetle (φ ij) from foreign ports in the countries with known beetle presence. (DOCX) [file pone.0044589.s002.docx]

Table S2. Ranking of Australian ports for receiving the Khapra beetle from foreign ports. Ports ranked by arrival rate of Khapra beetle (*ϕ _ij_*) from foreign ports in the countries with known beetle presence.

| **Australian Port** | ***ϕ_ij_*** | **relative *ϕ_ij_**** |
| --- | --- | --- |
| Melbourne | 0.5468195 | 8.9207194 |
| Botany Bay | 0.3976133 | 6.4865950 |
| Brisbane | 0.3904312 | 6.3694273 |
| Bell Bay | 0.2167836 | 3.5365693 |
| Fremantle | 0.1542654 | 2.5166590 |
| Adelaide | 0.0948990 | 1.5481657 |
| Burnie | 0.0495321 | 0.8080579 |
| Sydney | 0.0256229 | 0.4180071 |
| Hobart | 0.0137925 | 0.2250078 |
| Newcastle | 0.0029091 | 0.0474581 |
| Port Kembla | 0.0019770 | 0.0322521 |
| Gove | 0.0011788 | 0.0192313 |
| Darwin | 0.0010695 | 0.0174480 |
| Devonport | 0.0010211 | 0.0166579 |
| Townsville | 0.0007663 | 0.0125008 |
| Gladstone | 0.0003115 | 0.0050810 |
| Geelong | 0.0002875 | 0.0046896 |
| Hastings | 0.0002180 | 0.0035561 |
| Port Alma | 0.0002170 | 0.0035398 |
| Port Walcott | 0.0001810 | 0.0029526 |
| Dampier | 0.0001180 | 0.0019249 |
| Mackay | 0.0001145 | 0.0018678 |
| Weipa | 0.0000440 | 0.0007178 |
| Bunbury | 0.0000400 | 0.0006525 |
| Port Hedland | 0.0000065 | 0.0001060 |
| Yamba | 0.0000060 | 0.0000979 |
| Portland | 0.0000020 | 0.0000326 |
| Karumba | 0.0000010 | 0.0000163 |
| Cairns | 0.0000005 | 0.0000082 |
| Geraldton | 0 | 0 |
| Esperance | 0 | 0 |
| **Mean** | **0.0612977** |  |

***** denotes the relative pest’s arrival rate versus the avergae *ϕ_ij_* values for all network locations ($\overline{\phi}_{ij}$ = 0.0613)
