# Supplementary material for: Modelling the Arrival of Invasive Organisms via the International Marine Shipping Network: A Khapra Beetle Study
Source: PLoS One. 2012 Sep 6;7(9):e44589. doi: 10.1371/journal.pone.0044589 (PMC3435288; doi:10.1371/journal.pone.0044589)
Supplement: Table S3 — Ranking of all source ports for Khapra beetle introduction to the Australian port of Melbourne. (DOCX) [file pone.0044589.s003.docx]

Table S3. Ranking of all source ports for Khapra beetle introduction to the Australian port of Melbourne.

| **Melbourne** |  |  |  |  |  |  |  |  |  |  |  |
| --- | --- | --- | --- | --- | --- | --- | --- | --- | --- | --- | --- |
| **Port of origin *i*** | **Country** | ***ϕ_ij_*** | **relative *ϕ_ij_**** | **Port of origin *i*** | **Country** | ***ϕ_ij_*** | **relative *ϕ_ij_**** | **Port of origin *i*** | **Country** | ***ϕ_ij_*** | **relative *ϕ_ij_**** |
| Busan | KOR | 0.2453320 | 94.60130 | Limassol | CYP | 0.0006685 | 0.25778 | Ain Sukhna Term. | EGY | 0.0000245 | 0.00945 |
| Kaohsiung | TWN | 0.2270000 | 87.53239 | Ambarli | TUR | 0.0006450 | 0.24872 | Malaga | ESP | 0.0000135 | 0.00521 |
| Keelung | TWN | 0.0749780 | 28.91191 | Bilbao | ESP | 0.0006245 | 0.24081 | Pasajes | ESP | 0.0000125 | 0.00482 |
| Damietta | EGY | 0.0387065 | 14.92543 | Istanbul | TUR | 0.0005905 | 0.22770 | Mai-Liao | TWN | 0.0000110 | 0.00424 |
| Colombo | LKA | 0.0208125 | 8.02541 | Mumbai | IND | 0.0004950 | 0.19087 | Yanbu | SAU | 0.0000085 | 0.00328 |
| Jeddah | SAU | 0.0185600 | 7.15683 | New Tuticorin | IND | 0.0004805 | 0.18528 | Jubail | SAU | 0.0000080 | 0.00308 |
| Valencia | ESP | 0.0179365 | 6.91641 | Izmir | TUR | 0.0004795 | 0.18490 | Mongla | BGD | 0.0000080 | 0.00308 |
| Ulsan | KOR | 0.0168835 | 6.51037 | Ashkelon | ISR | 0.0003970 | 0.15309 | Tuzla | TUR | 0.0000070 | 0.00270 |
| Port Said | EGY | 0.0107895 | 4.16049 | Yarimca | TUR | 0.0003790 | 0.14614 | Nouakchott | MRT | 0.0000070 | 0.00270 |
| Barcelona | ESP | 0.0066630 | 2.56929 | Suez | EGY | 0.0003680 | 0.14190 | Eilat | ISR | 0.0000050 | 0.00193 |
| Gwangyang | KOR | 0.0057465 | 2.21588 | Alexandria | EGY | 0.0003540 | 0.13650 | Algiers | DZA | 0.0000035 | 0.00135 |
| Algeciras | ESP | 0.0041430 | 1.59756 | Gemlik | TUR | 0.0003325 | 0.12821 | Mundra | IND | 0.0000030 | 0.00116 |
| Aden | YEM | 0.0029465 | 1.13619 | Yosu | KOR | 0.0003265 | 0.12590 | Santander | ESP | 0.0000015 | 0.00058 |
| Jawaharlal Nehru | IND | 0.0028640 | 1.10437 | Montevideo | URY | 0.0002960 | 0.11414 | Samho | KOR | 0.0000010 | 0.00039 |
| Taichung | TWN | 0.0024035 | 0.92680 | Beirut | LBN | 0.0002610 | 0.10064 | Bandirma | TUR | 0.0000005 | 0.00019 |
| Dammam | SAU | 0.0022440 | 0.86530 | Cadiz | ESP | 0.0002025 | 0.07809 | Ceuta | ESP | 0.0000005 | 0.00019 |
| Masan | KOR | 0.0017755 | 0.68464 | Visakhapatnam | IND | 0.0001770 | 0.06825 | Mukalla | YEM | 0 | 0 |
| Karachi | PAK | 0.0017660 | 0.68098 | Chittagong | BGD | 0.0001640 | 0.06324 | Ras Lanuf | LBY | 0 | 0 |
| Chennai | IND | 0.0017560 | 0.67712 | Kolkata | IND | 0.0001015 | 0.03914 | Pyeongtaek | KOR | 0 | 0 |
| Incheon | KOR | 0.0012730 | 0.49088 | Kandla | IND | 0.0000940 | 0.03625 | Donghae | KOR | 0 | 0 |
| Bandar Abbas | IRN | 0.0011800 | 0.45501 | Kochi | IND | 0.0000885 | 0.03413 | Lattakia | SYR | 0 | 0 |
| Mersin | TUR | 0.0010690 | 0.41221 | Tripoli | LBY | 0.0000800 | 0.03085 | Alang | IND | 0 | 0 |
| Apapa-Lagos | NGA | 0.0010510 | 0.40527 | Arzew | DZA | 0.0000680 | 0.02622 | Karwar | IND | 0 | 0 |
| El Dekheila | EGY | 0.0007785 | 0.30019 | Derince | TUR | 0.0000650 | 0.02506 | Sikka | IND | 0 | 0 |
| Port Sudan | SDN | 0.0007315 | 0.28207 | Kakinada | IND | 0.0000590 | 0.02275 | Onne | NGA | 0 | 0 |
| Port Muhammad Bin Qasim | PAK | 0.0007240 | 0.27918 | Palma | ESP | 0.0000575 | 0.02217 | Dakar | SEN | 0 | 0 |
| Hodeidah | YEM | 0.0007015 | 0.27050 | Haldia | IND | 0.0000495 | 0.01909 | Casablanca | MAR | 0 | 0 |
| Haifa | ISR | 0.0006945 | 0.26780 | Sokhna | EGY | 0.0000420 | 0.01620 | Motril | ESP | 0 | 0 |
| Ashdod | ISR | 0.0006710 | 0.25874 | Tarragona | ESP | 0.0000415 | 0.01600 | Seville | ESP | 0 | 0 |

***** denotes the relative pest’s arrival rate versus the avergae *ϕ_ij_* values for all network locations (i.e. the mean of all *ϕ_ij_* values in Tables S3-S12)

($\overline{\phi}_{ij}$= 0.00259)
