# Supplementary material for: Modelling the Arrival of Invasive Organisms via the International Marine Shipping Network: A Khapra Beetle Study
Source: PLoS One. 2012 Sep 6;7(9):e44589. doi: 10.1371/journal.pone.0044589 (PMC3435288; doi:10.1371/journal.pone.0044589)
Supplement: Table S4 — Ranking of all source ports for Khapra beetle introduction to the Australian port of Botany Bay. (DOCX) [file pone.0044589.s004.docx]

Table S4. Ranking of all source ports for Khapra beetle introduction to the Australian port of Botany Bay.

| **Botany Bay (Sydney)** |  |  |  |  |  |  |  |  |  |  |  |
| --- | --- | --- | --- | --- | --- | --- | --- | --- | --- | --- | --- |
| **Port of origin *i*** | **Country** | ***ϕ_ij_*** | **relative *ϕ_ij_**** | **Port of origin *i*** | **Country** | ***ϕ_ij_*** | **relative *ϕ_ij_**** | **Port of origin *i*** | **Country** | ***ϕ_ij_*** | **relative *ϕ_ij_**** |
| Kaohsiung | TWN | 0.1594200 | 61.47318 | Port Sudan | SDN | 0.0004350 | 0.16774 | Tripoli | LBY | 0.0000250 | 0.00964 |
| Busan | KOR | 0.1582450 | 61.02010 | Bilbao | ESP | 0.0004220 | 0.16273 | Pasajes | ESP | 0.0000110 | 0.00424 |
| Keelung | TWN | 0.0528270 | 20.37037 | Ambarli | TUR | 0.0004090 | 0.15771 | Eilat | ISR | 0.0000090 | 0.00347 |
| Damietta | EGY | 0.0251605 | 9.70202 | Istanbul | TUR | 0.0003840 | 0.14807 | Mai-Liao | TWN | 0.0000080 | 0.00308 |
| Colombo | LKA | 0.0130410 | 5.02868 | New Tuticorin | IND | 0.0003455 | 0.13323 | Jubail | SAU | 0.0000065 | 0.00251 |
| Jeddah | SAU | 0.0121995 | 4.70419 | Yarimca | TUR | 0.0003070 | 0.11838 | Sokhna | EGY | 0.0000050 | 0.00193 |
| Valencia | ESP | 0.0119115 | 4.59314 | Izmir | TUR | 0.0002890 | 0.11144 | Yanbu | SAU | 0.0000045 | 0.00174 |
| Port Said | EGY | 0.0069330 | 2.67340 | Mumbai | IND | 0.0002795 | 0.10778 | Tuzla | TUR | 0.0000045 | 0.00174 |
| Barcelona | ESP | 0.0045050 | 1.73715 | Alexandria | EGY | 0.0002520 | 0.09717 | Nouakchott | MRT | 0.0000040 | 0.00154 |
| Ulsan | KOR | 0.0038910 | 1.50039 | Ashkelon | ISR | 0.0002505 | 0.09659 | Mongla | BGD | 0.0000035 | 0.00135 |
| Gwangyang | KOR | 0.0037305 | 1.43850 | Montevideo | URY | 0.0002415 | 0.09312 | Bandirma | TUR | 0.0000030 | 0.00116 |
| Algeciras | ESP | 0.0027970 | 1.07854 | Suez | EGY | 0.0002365 | 0.09120 | Algiers | DZA | 0.0000020 | 0.00077 |
| Jawaharlal Nehru | IND | 0.0022665 | 0.87397 | Gemlik | TUR | 0.0002125 | 0.08194 | Mundra | IND | 0.0000015 | 0.00058 |
| Aden | YEM | 0.0017740 | 0.68406 | Yosu | KOR | 0.0002100 | 0.08098 | Samho | KOR | 0.0000010 | 0.00039 |
| Taichung | TWN | 0.0016185 | 0.62410 | Beirut | LBN | 0.0001625 | 0.06266 | Ceuta | ESP | 0.0000005 | 0.00019 |
| Dammam | SAU | 0.0014595 | 0.56279 | Cadiz | ESP | 0.0001060 | 0.04087 | Mukalla | YEM | 0 | 0 |
| Chennai | IND | 0.0012015 | 0.46330 | Chittagong | BGD | 0.0000945 | 0.03644 | Santander | ESP | 0 | 0 |
| Karachi | PAK | 0.0011920 | 0.45964 | Visakhapatnam | IND | 0.0000860 | 0.03316 | Ras Lanuf | LBY | 0 | 0 |
| Masan | KOR | 0.0011635 | 0.44865 | Kandla | IND | 0.0000675 | 0.02603 | Pyeongtaek | KOR | 0 | 0 |
| Incheon | KOR | 0.0009235 | 0.35611 | Kochi | IND | 0.0000635 | 0.02449 | Donghae | KOR | 0 | 0 |
| Bandar Abbas | IRN | 0.0007375 | 0.28438 | Kolkata | IND | 0.0000535 | 0.02063 | Lattakia | SYR | 0 | 0 |
| Mersin | TUR | 0.0006605 | 0.25469 | Ain Sukhna Term. | EGY | 0.0000500 | 0.01928 | Alang | IND | 0 | 0 |
| Apapa-Lagos | NGA | 0.0006470 | 0.24949 | Malaga | ESP | 0.0000450 | 0.01735 | Karwar | IND | 0 | 0 |
| Ashdod | ISR | 0.0005245 | 0.20225 | Palma | ESP | 0.0000360 | 0.01388 | Sikka | IND | 0 | 0 |
| Port Muhammad Bin Qasim | PAK | 0.0005210 | 0.20090 | Arzew | DZA | 0.0000335 | 0.01292 | Onne | NGA | 0 | 0 |
| El Dekheila | EGY | 0.0004940 | 0.19049 | Derince | TUR | 0.0000325 | 0.01253 | Dakar | SEN | 0 | 0 |
| Hodeidah | YEM | 0.0004780 | 0.18432 | Haldia | IND | 0.0000315 | 0.01215 | Casablanca | MAR | 0 | 0 |
| Haifa | ISR | 0.0004755 | 0.18336 | Kakinada | IND | 0.0000265 | 0.01022 | Motril | ESP | 0 | 0 |
| Limassol | CYP | 0.0004520 | 0.17429 | Tarragona | ESP | 0.0000250 | 0.00964 | Seville | ESP | 0 | 0 |

***** denotes the relative pest’s arrival rate versus the avergae *ϕ_ij_* values for all network locations (i.e. the mean of all *ϕ_ij_* values in Tables S3-S12) ($\overline{\phi}_{ij}$ = 0.00259)
