# Supplementary material for: Modelling the Arrival of Invasive Organisms via the International Marine Shipping Network: A Khapra Beetle Study
Source: PLoS One. 2012 Sep 6;7(9):e44589. doi: 10.1371/journal.pone.0044589 (PMC3435288; doi:10.1371/journal.pone.0044589)
Supplement: Table S5 — Ranking of all source ports for Khapra beetle introduction to the Australian port of Brisbane. (DOCX) [file pone.0044589.s005.docx]

Table S5. Ranking of all source ports for Khapra beetle introduction to the Australian port of Brisbane.

| **Brisbane** |  |  |  |  |  |  |  |  |  |  |  |
| --- | --- | --- | --- | --- | --- | --- | --- | --- | --- | --- | --- |
| **Port of origin *i*** | **Country** | ***ϕ_ij_*** | **relative *ϕ_ij_**** | **Port of origin *i*** | **Country** | ***ϕ_ij_*** | **relative *ϕ_ij_**** | **Port of origin *i*** | **Country** | ***ϕ_ij_*** | **relative *ϕ_ij_**** |
| Busan | KOR | 0.1595950 | 366830.29851 | Haifa | ISR | 0.0004110 | 944.68657 | Tarragona | ESP | 0.0000265 | 60.91045 |
| Kaohsiung | TWN | 0.1584480 | 364193.91045 | New Tuticorin | IND | 0.0003730 | 857.34328 | Ain Sukhna Term. | EGY | 0.0000245 | 56.31343 |
| Keelung | TWN | 0.0522620 | 120124.59701 | Yarimca | TUR | 0.0003695 | 849.29851 | Mongla | BGD | 0.0000085 | 19.53731 |
| Damietta | EGY | 0.0234515 | 53903.44776 | Istanbul | TUR | 0.0003410 | 783.79104 | Nouakchott | MRT | 0.0000085 | 19.53731 |
| Colombo | LKA | 0.0115480 | 26543.16418 | Bilbao | ESP | 0.0003315 | 761.95522 | Pasajes | ESP | 0.0000080 | 18.38806 |
| Jeddah | SAU | 0.0096345 | 22144.97015 | Ambarli | TUR | 0.0003110 | 714.83582 | Malaga | ESP | 0.0000070 | 16.08955 |
| Valencia | ESP | 0.0087870 | 20196.98507 | Ashkelon | ISR | 0.0003005 | 690.70149 | Tuzla | TUR | 0.0000065 | 14.94030 |
| Port Said | EGY | 0.0054050 | 12423.43284 | Mumbai | IND | 0.0002540 | 583.82090 | Eilat | ISR | 0.0000045 | 10.34328 |
| Gwangyang | KOR | 0.0041785 | 9604.31343 | Yosu | KOR | 0.0002390 | 549.34328 | Yanbu | SAU | 0.0000030 | 6.89552 |
| Ulsan | KOR | 0.0038815 | 8921.65672 | Montevideo | URY | 0.0002230 | 512.56716 | Sokhna | EGY | 0.0000025 | 5.74627 |
| Barcelona | ESP | 0.0035510 | 8162.00000 | Alexandria | EGY | 0.0002075 | 476.94030 | Bandirma | TUR | 0.0000020 | 4.59701 |
| Aden | YEM | 0.0026040 | 5985.31343 | Izmir | TUR | 0.0002065 | 474.64179 | Samho | KOR | 0.0000010 | 2.29851 |
| Algeciras | ESP | 0.0023015 | 5290.01493 | Suez | EGY | 0.0002025 | 465.44776 | Santander | ESP | 0.0000010 | 2.29851 |
| Jawaharlal Nehru | IND | 0.0021695 | 4986.61194 | Beirut | LBN | 0.0001630 | 374.65672 | Mundra | IND | 0.0000005 | 1.14925 |
| Dammam | SAU | 0.0014890 | 3422.47761 | Gemlik | TUR | 0.0001515 | 348.22388 | Algiers | DZA | 0.0000005 | 1.14925 |
| Taichung | TWN | 0.0013300 | 3057.01493 | Chittagong | BGD | 0.0001445 | 332.13433 | Ceuta | ESP | 0.0000005 | 1.14925 |
| Karachi | PAK | 0.0012760 | 2932.89552 | Cadiz | ESP | 0.0001180 | 271.22388 | Mukalla | YEM | 0 | 0 |
| Masan | KOR | 0.0011040 | 2537.55224 | Kochi | IND | 0.0001155 | 265.47761 | Ras Lanuf | LBY | 0 | 0 |
| Chennai | IND | 0.0009095 | 2090.49254 | Visakhapatnam | IND | 0.0001115 | 256.28358 | Pyeongtaek | KOR | 0 | 0 |
| Incheon | KOR | 0.0009080 | 2087.04478 | Kolkata | IND | 0.0001050 | 241.34328 | Donghae | KOR | 0 | 0 |
| Bandar Abbas | IRN | 0.0006665 | 1531.95522 | Kandla | IND | 0.0000775 | 178.13433 | Lattakia | SYR | 0 | 0 |
| Mersin | TUR | 0.0006610 | 1519.31343 | Palma | ESP | 0.0000705 | 162.04478 | Alang | IND | 0 | 0 |
| Hodeidah | YEM | 0.0005765 | 1325.08955 | Derince | TUR | 0.0000600 | 137.91045 | Karwar | IND | 0 | 0 |
| Port Sudan | SDN | 0.0004965 | 1141.20896 | Mai-Liao | TWN | 0.0000485 | 111.47761 | Sikka | IND | 0 | 0 |
| Apapa-Lagos | NGA | 0.0004935 | 1134.31343 | Arzew | DZA | 0.0000400 | 91.94030 | Onne | NGA | 0 | 0 |
| Ashdod | ISR | 0.0004915 | 1129.71642 | Jubail | SAU | 0.0000390 | 89.64179 | Dakar | SEN | 0 | 0 |
| El Dekheila | EGY | 0.0004745 | 1090.64179 | Kakinada | IND | 0.0000315 | 72.40299 | Casablanca | MAR | 0 | 0 |
| Limassol | CYP | 0.0004475 | 1028.58209 | Tripoli | LBY | 0.0000295 | 67.80597 | Motril | ESP | 0 | 0 |
| Port Muhammad Bin Qasim | PAK | 0.0004200 | 965.37313 | Haldia | IND | 0.0000270 | 62.05970 | Seville | ESP | 0 | 0 |

***** denotes the relative pest’s arrival rate versus the avergae *ϕ_ij_* values for all network locations (i.e. the mean of all *ϕ_ij_* values in Tables S3-S12) ($\overline{\phi}_{ij}$ = 0.00259)
