# Supplementary material for: Modelling the Arrival of Invasive Organisms via the International Marine Shipping Network: A Khapra Beetle Study
Source: PLoS One. 2012 Sep 6;7(9):e44589. doi: 10.1371/journal.pone.0044589 (PMC3435288; doi:10.1371/journal.pone.0044589)
Supplement: Table S6 — Ranking of all source ports for Khapra beetle introduction to the Australian port of Bell Bay. (DOCX) [file pone.0044589.s006.docx]

Table S6. Ranking of all source ports for Khapra beetle introduction to the Australian port of Bell Bay.

| **Bell Bay** |  |  |  |  |  |  |  |  |  |  |  |
| --- | --- | --- | --- | --- | --- | --- | --- | --- | --- | --- | --- |
| **Port of origin *i*** | **Country** | ***ϕ_ij_*** | **relative *ϕ_ij_**** | **Port of origin *i*** | **Country** | ***ϕ_ij_*** | **relative *ϕ_ij_**** | **Port of origin *i*** | **Country** | ***ϕ_ij_*** | **relative *ϕ_ij_**** |
| Busan | KOR | 0.0838840 | 192808.00000 | Ashdod | ISR | 0.0002095 | 481.53731 | Ain Sukhna Term. | EGY | 0.0000055 | 12.64179 |
| Kaohsiung | TWN | 0.0704900 | 162021.79104 | Limassol | CYP | 0.0002075 | 476.94030 | Malaga | ESP | 0.0000050 | 11.49254 |
| Keelung | TWN | 0.0232015 | 53328.82090 | Bilbao | ESP | 0.0002045 | 470.04478 | Pasajes | ESP | 0.0000040 | 9.19403 |
| Damietta | EGY | 0.0121385 | 27900.43284 | Mumbai | IND | 0.0001860 | 427.52239 | Mai-Liao | TWN | 0.0000040 | 9.19403 |
| Ulsan | KOR | 0.0099830 | 22946.00000 | Istanbul | TUR | 0.0001820 | 418.32836 | Nouakchott | MRT | 0.0000035 | 8.04478 |
| Colombo | LKA | 0.0065110 | 14965.58209 | New Tuticorin | IND | 0.0001560 | 358.56716 | Yanbu | SAU | 0.0000025 | 5.74627 |
| Jeddah | SAU | 0.0059830 | 13751.97015 | Izmir | TUR | 0.0001545 | 355.11940 | Mongla | BGD | 0.0000025 | 5.74627 |
| Valencia | ESP | 0.0057930 | 13315.25373 | Ashkelon | ISR | 0.0001350 | 310.29851 | Jubail | SAU | 0.0000020 | 4.59701 |
| Port Said | EGY | 0.0033045 | 7595.41791 | Yarimca | TUR | 0.0001235 | 283.86567 | Tuzla | TUR | 0.0000020 | 4.59701 |
| Barcelona | ESP | 0.0020815 | 4784.34328 | Suez | EGY | 0.0001125 | 258.58209 | Eilat | ISR | 0.0000010 | 2.29851 |
| Gwangyang | KOR | 0.0019605 | 4506.22388 | Gemlik | TUR | 0.0001100 | 252.83582 | Algiers | DZA | 0.0000010 | 2.29851 |
| Algeciras | ESP | 0.0012865 | 2957.02985 | Alexandria | EGY | 0.0001095 | 251.68657 | Mundra | IND | 0.0000005 | 1.14925 |
| Aden | YEM | 0.0011750 | 2700.74627 | Yosu | KOR | 0.0000950 | 218.35821 | Santander | ESP | 0.0000005 | 1.14925 |
| Jawaharlal Nehru | IND | 0.0009720 | 2234.14925 | Montevideo | URY | 0.0000925 | 212.61194 | Bandirma | TUR | 0 | 0 |
| Taichung | TWN | 0.0007920 | 1820.41791 | Beirut | LBN | 0.0000865 | 198.82090 | Mukalla | YEM | 0 | 0 |
| Dammam | SAU | 0.0007145 | 1642.28358 | Visakhapatnam | IND | 0.0000690 | 158.59701 | Samho | KOR | 0 | 0 |
| Chennai | IND | 0.0006020 | 1383.70149 | Cadiz | ESP | 0.0000600 | 137.91045 | Ceuta | ESP | 0 | 0 |
| Karachi | PAK | 0.0005835 | 1341.17910 | Chittagong | BGD | 0.0000540 | 124.11940 | Ras Lanuf | LBY | 0 | 0 |
| Masan | KOR | 0.0005640 | 1296.35821 | Kolkata | IND | 0.0000350 | 80.44776 | Pyeongtaek | KOR | 0 | 0 |
| Incheon | KOR | 0.0004010 | 921.70149 | Kochi | IND | 0.0000345 | 79.29851 | Donghae | KOR | 0 | 0 |
| Bandar Abbas | IRN | 0.0003980 | 914.80597 | Kandla | IND | 0.0000295 | 67.80597 | Lattakia | SYR | 0 | 0 |
| Apapa-Lagos | NGA | 0.0003550 | 815.97015 | Tripoli | LBY | 0.0000275 | 63.20896 | Alang | IND | 0 | 0 |
| Mersin | TUR | 0.0003430 | 788.38806 | Derince | TUR | 0.0000265 | 60.91045 | Karwar | IND | 0 | 0 |
| El Dekheila | EGY | 0.0002460 | 565.43284 | Arzew | DZA | 0.0000260 | 59.76119 | Sikka | IND | 0 | 0 |
| Port Muhammad Bin Qasim | PAK | 0.0002395 | 550.49254 | Kakinada | IND | 0.0000245 | 56.31343 | Onne | NGA | 0 | 0 |
| Port Sudan | SDN | 0.0002315 | 532.10448 | Haldia | IND | 0.0000200 | 45.97015 | Dakar | SEN | 0 | 0 |
| Haifa | ISR | 0.0002235 | 513.71642 | Palma | ESP | 0.0000195 | 44.82090 | Casablanca | MAR | 0 | 0 |
| Ambarli | TUR | 0.0002195 | 504.52239 | Tarragona | ESP | 0.0000135 | 31.02985 | Motril | ESP | 0 | 0 |
| Hodeidah | YEM | 0.0002150 | 494.17910 | Sokhna | EGY | 0.0000130 | 29.88060 | Seville | ESP | 0 | 0 |

***** denotes the relative pest’s arrival rate versus the avergae *ϕ_ij_* values for all network locations (i.e. the mean of all *ϕ_ij_* values in Tables S3-S12) ($\overline{\phi}_{ij}$ = 0.00259)
