# Supplementary material for: Modelling the Arrival of Invasive Organisms via the International Marine Shipping Network: A Khapra Beetle Study
Source: PLoS One. 2012 Sep 6;7(9):e44589. doi: 10.1371/journal.pone.0044589 (PMC3435288; doi:10.1371/journal.pone.0044589)
Supplement: Table S7 — Ranking of all source ports for Khapra beetle introduction to the Australian port of Fremantle. (DOCX) [file pone.0044589.s007.docx]

Table S7. Ranking of all source ports for Khapra beetle introduction to the Australian port of Fremantle.

| **Fremantle** |  |  |  |  |  |  |  |  |  |  |  |
| --- | --- | --- | --- | --- | --- | --- | --- | --- | --- | --- | --- |
| **Port of origin *i*** | **Country** | ***ϕ_ij_*** | **relative *ϕ_ij_**** | **Port of origin *i*** | **Country** | ***ϕ_ij_*** | **relative *ϕ_ij_**** | **Port of origin *i*** | **Country** | ***ϕ_ij_*** | **relative *ϕ_ij_**** |
| Busan | KOR | 0.0508600 | 116902.08955 | Port Muhammad Bin Qasim | PAK | 0.0002420 | 556.23881 | Ain Sukhna Term. | EGY | 0.0000080 | 18.38806 |
| Kaohsiung | TWN | 0.0424370 | 97541.76119 | Istanbul | TUR | 0.0002210 | 507.97015 | Mongla | BGD | 0.0000070 | 16.08955 |
| Damietta | EGY | 0.0120660 | 27733.79104 | Ashdod | ISR | 0.0002180 | 501.07463 | Yanbu | SAU | 0.0000045 | 10.34328 |
| Keelung | TWN | 0.0119650 | 27501.64179 | Gemlik | TUR | 0.0001885 | 433.26866 | Mai-Liao | TWN | 0.0000030 | 6.89552 |
| Valencia | ESP | 0.0087365 | 20080.91045 | Haifa | ISR | 0.0001855 | 426.37313 | Sokhna | EGY | 0.0000030 | 6.89552 |
| Colombo | LKA | 0.0075645 | 17387.05970 | Mumbai | IND | 0.0001745 | 401.08955 | Jubail | SAU | 0.0000025 | 5.74627 |
| Jeddah | SAU | 0.0073830 | 16969.88060 | Apapa-Lagos | NGA | 0.0001730 | 397.64179 | Malaga | ESP | 0.0000025 | 5.74627 |
| Port Said | EGY | 0.0042090 | 9674.41791 | Visakhapatnam | IND | 0.0001490 | 342.47761 | Eilat | ISR | 0.0000025 | 5.74627 |
| Barcelona | ESP | 0.0026285 | 6041.62687 | Suez | EGY | 0.0001435 | 329.83582 | Mundra | IND | 0.0000025 | 5.74627 |
| Gwangyang | KOR | 0.0021120 | 4854.44776 | Hodeidah | YEM | 0.0001245 | 286.16418 | Nouakchott | MRT | 0.0000015 | 3.44776 |
| Ulsan | KOR | 0.0016460 | 3783.34328 | Cadiz | ESP | 0.0001035 | 237.89552 | Samho | KOR | 0.0000015 | 3.44776 |
| Jawaharlal Nehru | IND | 0.0015365 | 3531.65672 | Alexandria | EGY | 0.0000980 | 225.25373 | Bandirma | TUR | 0.0000010 | 2.29851 |
| Algeciras | ESP | 0.0014915 | 3428.22388 | Yarimca | TUR | 0.0000935 | 214.91045 | Algiers | DZA | 0.0000005 | 1.14925 |
| Aden | YEM | 0.0013055 | 3000.70149 | Haldia | IND | 0.0000860 | 197.67164 | Tuzla | TUR | 0 | 0 |
| Dammam | SAU | 0.0009410 | 2162.89552 | Chittagong | BGD | 0.0000835 | 191.92537 | Mukalla | YEM | 0 | 0 |
| Bandar Abbas | IRN | 0.0006760 | 1553.79104 | Montevideo | URY | 0.0000600 | 137.91045 | Santander | ESP | 0 | 0 |
| Taichung | TWN | 0.0005735 | 1318.19403 | Kakinada | IND | 0.0000535 | 122.97015 | Ceuta | ESP | 0 | 0 |
| Karachi | PAK | 0.0005505 | 1265.32836 | Ashkelon | ISR | 0.0000520 | 119.52239 | Ras Lanuf | LBY | 0 | 0 |
| Masan | KOR | 0.0005080 | 1167.64179 | Tripoli | LBY | 0.0000495 | 113.77612 | Pyeongtaek | KOR | 0 | 0 |
| Chennai | IND | 0.0004770 | 1096.38806 | Yosu | KOR | 0.0000430 | 98.83582 | Donghae | KOR | 0 | 0 |
| Port Sudan | SDN | 0.0003920 | 901.01493 | Kolkata | IND | 0.0000430 | 98.83582 | Lattakia | SYR | 0 | 0 |
| El Dekheila | EGY | 0.0002980 | 684.95522 | Kandla | IND | 0.0000370 | 85.04478 | Alang | IND | 0 | 0 |
| Bilbao | ESP | 0.0002900 | 666.56716 | Beirut | LBN | 0.0000320 | 73.55224 | Karwar | IND | 0 | 0 |
| Incheon | KOR | 0.0002795 | 642.43284 | Pasajes | ESP | 0.0000320 | 73.55224 | Sikka | IND | 0 | 0 |
| New Tuticorin | IND | 0.0002590 | 595.31343 | Kochi | IND | 0.0000280 | 64.35821 | Onne | NGA | 0 | 0 |
| Limassol | CYP | 0.0002540 | 583.82090 | Derince | TUR | 0.0000255 | 58.61194 | Dakar | SEN | 0 | 0 |
| Ambarli | TUR | 0.0002520 | 579.22388 | Arzew | DZA | 0.0000220 | 50.56716 | Casablanca | MAR | 0 | 0 |
| Izmir | TUR | 0.0002475 | 568.88060 | Palma | ESP | 0.0000220 | 50.56716 | Motril | ESP | 0 | 0 |
| Mersin | TUR | 0.0002420 | 556.23881 | Tarragona | ESP | 0.0000185 | 42.52239 | Seville | ESP | 0 | 0 |

***** denotes the relative pest’s arrival rate versus the avergae *ϕ_ij_* values for all network locations (i.e. the mean of all *ϕ_ij_* values in Tables S3-S12) ($\overline{\phi}_{ij}$ = 0.00259)
