# Supplementary material for: Modelling the Arrival of Invasive Organisms via the International Marine Shipping Network: A Khapra Beetle Study
Source: PLoS One. 2012 Sep 6;7(9):e44589. doi: 10.1371/journal.pone.0044589 (PMC3435288; doi:10.1371/journal.pone.0044589)
Supplement: Table S8 — Ranking of all source ports for Khapra beetle introduction to the Australian port of Adelaide. (DOCX) [file pone.0044589.s008.docx]

Table S8. Ranking of all source ports for Khapra beetle introduction to the Australian port of Adelaide.

| **Adelaide** |  |  |  |  |  |  |  |  |  |  |  |
| --- | --- | --- | --- | --- | --- | --- | --- | --- | --- | --- | --- |
| **Port of origin *i*** | **Country** | ***ϕ_ij_*** | **relative *ϕ_ij_**** | **Port of origin *i*** | **Country** | ***ϕ_ij_*** | **relative *ϕ_ij_**** | **Port of origin *i*** | **Country** | ***ϕ_ij_*** | **relative *ϕ_ij_**** |
| Busan | KOR | 0.0287645 | 66115.41791 | Istanbul | TUR | 0.0001320 | 303.40299 | Malaga | ESP | 0.0000045 | 10.34328 |
| Kaohsiung | TWN | 0.0239790 | 55115.91045 | Port Muhammad Bin Qasim | PAK | 0.0001250 | 287.31343 | Pasajes | ESP | 0.0000040 | 9.19403 |
| Damietta | EGY | 0.0092280 | 21210.62687 | Haifa | ISR | 0.0001155 | 265.47761 | Yanbu | SAU | 0.0000030 | 6.89552 |
| Keelung | TWN | 0.0069460 | 15965.43284 | Ashdod | ISR | 0.0001110 | 255.13433 | Eilat | ISR | 0.0000020 | 4.59701 |
| Colombo | LKA | 0.0056965 | 13093.44776 | Suez | EGY | 0.0001095 | 251.68657 | Jubail | SAU | 0.0000015 | 3.44776 |
| Jeddah | SAU | 0.0052435 | 12052.22388 | Apapa-Lagos | NGA | 0.0001015 | 233.29851 | Sokhna | EGY | 0.0000015 | 3.44776 |
| Valencia | ESP | 0.0049775 | 11440.82090 | Kandla | IND | 0.0000810 | 186.17910 | Mai-Liao | TWN | 0.0000010 | 2.29851 |
| Port Said | EGY | 0.0032930 | 7568.98507 | Gemlik | TUR | 0.0000700 | 160.89552 | Nouakchott | MRT | 0.0000010 | 2.29851 |
| Barcelona | ESP | 0.0015950 | 3666.11940 | Hodeidah | YEM | 0.0000620 | 142.50746 | Mundra | IND | 0.0000010 | 2.29851 |
| Algeciras | ESP | 0.0011510 | 2645.58209 | New Tuticorin | IND | 0.0000615 | 141.35821 | Samho | KOR | 0.0000010 | 2.29851 |
| Gwangyang | KOR | 0.0010540 | 2422.62687 | Yarimca | TUR | 0.0000595 | 136.76119 | Bandirma | TUR | 0.0000005 | 1.14925 |
| Ulsan | KOR | 0.0009120 | 2096.23881 | Cadiz | ESP | 0.0000595 | 136.76119 | Mongla | BGD | 0.0000005 | 1.14925 |
| Aden | YEM | 0.0005870 | 1349.22388 | Alexandria | EGY | 0.0000570 | 131.01493 | Algiers | DZA | 0.0000005 | 1.14925 |
| Jawaharlal Nehru | IND | 0.0005160 | 1186.02985 | Ashkelon | ISR | 0.0000340 | 78.14925 | Tuzla | TUR | 0 | 0 |
| Dammam | SAU | 0.0004090 | 940.08955 | Chittagong | BGD | 0.0000315 | 72.40299 | Mukalla | YEM | 0 | 0 |
| Bandar Abbas | IRN | 0.0003680 | 845.85075 | Yosu | KOR | 0.0000305 | 70.10448 | Santander | ESP | 0 | 0 |
| Masan | KOR | 0.0003225 | 741.26866 | Visakhapatnam | IND | 0.0000290 | 66.65672 | Ceuta | ESP | 0 | 0 |
| Karachi | PAK | 0.0003055 | 702.19403 | Montevideo | URY | 0.0000275 | 63.20896 | Ras Lanuf | LBY | 0 | 0 |
| Chennai | IND | 0.0002775 | 637.83582 | Kolkata | IND | 0.0000270 | 62.05970 | Pyeongtaek | KOR | 0 | 0 |
| Taichung | TWN | 0.0002260 | 519.46269 | Haldia | IND | 0.0000165 | 37.92537 | Donghae | KOR | 0 | 0 |
| Port Sudan | SDN | 0.0002170 | 498.77612 | Kochi | IND | 0.0000145 | 33.32836 | Lattakia | SYR | 0 | 0 |
| El Dekheila | EGY | 0.0001895 | 435.56716 | Beirut | LBN | 0.0000135 | 31.02985 | Alang | IND | 0 | 0 |
| Incheon | KOR | 0.0001880 | 432.11940 | Tripoli | LBY | 0.0000120 | 27.58209 | Karwar | IND | 0 | 0 |
| Bilbao | ESP | 0.0001835 | 421.77612 | Kakinada | IND | 0.0000120 | 27.58209 | Sikka | IND | 0 | 0 |
| Limassol | CYP | 0.0001765 | 405.68657 | Arzew | DZA | 0.0000115 | 26.43284 | Onne | NGA | 0 | 0 |
| Ambarli | TUR | 0.0001700 | 390.74627 | Palma | ESP | 0.0000095 | 21.83582 | Dakar | SEN | 0 | 0 |
| Mersin | TUR | 0.0001590 | 365.46269 | Tarragona | ESP | 0.0000095 | 21.83582 | Casablanca | MAR | 0 | 0 |
| Izmir | TUR | 0.0001425 | 327.53731 | Derince | TUR | 0.0000090 | 20.68657 | Motril | ESP | 0 | 0 |
| Mumbai | IND | 0.0001385 | 318.34328 | Ain Sukhna Term. | EGY | 0.0000055 | 12.64179 | Seville | ESP | 0 | 0 |

***** denotes the relative pest’s arrival rate versus the avergae *ϕ_ij_* values for all network locations (i.e. the mean of all *ϕ_ij_* values in Tables S3-S12) ($\overline{\phi}_{ij}$ = 0.00259)
