# Supplementary material for: Modelling the Arrival of Invasive Organisms via the International Marine Shipping Network: A Khapra Beetle Study
Source: PLoS One. 2012 Sep 6;7(9):e44589. doi: 10.1371/journal.pone.0044589 (PMC3435288; doi:10.1371/journal.pone.0044589)
Supplement: Table S9 — Ranking of all source ports for Khapra beetle introduction to the Australian port of Burnie. (DOCX) [file pone.0044589.s009.docx]

Table S9. Ranking of all source ports for Khapra beetle introduction to the Australian port of Burnie.

| **Burnie** |  |  |  |  |  |  |  |  |  |  |  |
| --- | --- | --- | --- | --- | --- | --- | --- | --- | --- | --- | --- |
| **Port of origin *i*** | **Country** | ***ϕ_ij_*** | **relative *ϕ_ij_**** | **Port of origin *i*** | **Country** | ***ϕ_ij_*** | **relative *ϕ_ij_**** | **Port of origin *i*** | **Country** | ***ϕ_ij_*** | **relative *ϕ_ij_**** |
| Busan | KOR | 0.0177015 | 40687.02985 | Istanbul | TUR | 0.0000440 | 101.13433 | Haldia | IND | 0.0000015 | 3.44776 |
| Kaohsiung | TWN | 0.0149445 | 34350.04478 | Hodeidah | YEM | 0.0000435 | 99.98507 | Ain Sukhna Term. | EGY | 0.0000015 | 3.44776 |
| Keelung | TWN | 0.0048660 | 11184.53731 | Limassol | CYP | 0.0000385 | 88.49254 | Mai-Liao | TWN | 0.0000015 | 3.44776 |
| Damietta | EGY | 0.0025725 | 5912.91045 | Ambarli | TUR | 0.0000375 | 86.19403 | Algiers | DZA | 0.0000010 | 2.29851 |
| Ulsan | KOR | 0.0021000 | 4826.86567 | Mumbai | IND | 0.0000345 | 79.29851 | Pasajes | ESP | 0.0000005 | 1.14925 |
| Colombo | LKA | 0.0013890 | 3192.62687 | Izmir | TUR | 0.0000320 | 73.55224 | Yanbu | SAU | 0.0000005 | 1.14925 |
| Jeddah | SAU | 0.0012410 | 2852.44776 | New Tuticorin | IND | 0.0000295 | 67.80597 | Nouakchott | MRT | 0.0000005 | 1.14925 |
| Valencia | ESP | 0.0012060 | 2772.00000 | Yarimca | TUR | 0.0000255 | 58.61194 | Eilat | ISR | 0.0000005 | 1.14925 |
| Port Said | EGY | 0.0006720 | 1544.59701 | Ashkelon | ISR | 0.0000240 | 55.16418 | Malaga | ESP | 0 | 0 |
| Barcelona | ESP | 0.0004475 | 1028.58209 | Gemlik | TUR | 0.0000230 | 52.86567 | Bandirma | TUR | 0 | 0 |
| Gwangyang | KOR | 0.0003970 | 912.50746 | Yosu | KOR | 0.0000210 | 48.26866 | Tuzla | TUR | 0 | 0 |
| Taichung | TWN | 0.0003950 | 907.91045 | Montevideo | URY | 0.0000210 | 48.26866 | Mukalla | YEM | 0 | 0 |
| Chennai | IND | 0.0003105 | 713.68657 | Alexandria | EGY | 0.0000205 | 47.11940 | Mongla | BGD | 0 | 0 |
| Algeciras | ESP | 0.0002655 | 610.25373 | Suez | EGY | 0.0000185 | 42.52239 | Mundra | IND | 0 | 0 |
| Aden | YEM | 0.0002460 | 565.43284 | Beirut | LBN | 0.0000160 | 36.77612 | Samho | KOR | 0 | 0 |
| Jawaharlal Nehru | IND | 0.0001935 | 444.76119 | Cadiz | ESP | 0.0000150 | 34.47761 | Santander | ESP | 0 | 0 |
| Dammam | SAU | 0.0001510 | 347.07463 | Chittagong | BGD | 0.0000120 | 27.58209 | Ceuta | ESP | 0 | 0 |
| Karachi | PAK | 0.0001350 | 310.29851 | Visakhapatnam | IND | 0.0000120 | 27.58209 | Ras Lanuf | LBY | 0 | 0 |
| Masan | KOR | 0.0001170 | 268.92537 | Tripoli | LBY | 0.0000095 | 21.83582 | Pyeongtaek | KOR | 0 | 0 |
| Apapa-Lagos | NGA | 0.0000940 | 216.05970 | Derince | TUR | 0.0000085 | 19.53731 | Donghae | KOR | 0 | 0 |
| Incheon | KOR | 0.0000825 | 189.62687 | Kolkata | IND | 0.0000080 | 18.38806 | Lattakia | SYR | 0 | 0 |
| Bandar Abbas | IRN | 0.0000775 | 178.13433 | Arzew | DZA | 0.0000075 | 17.23881 | Alang | IND | 0 | 0 |
| Mersin | TUR | 0.0000630 | 144.80597 | Palma | ESP | 0.0000070 | 16.08955 | Karwar | IND | 0 | 0 |
| Port Muhammad Bin Qasim | PAK | 0.0000550 | 126.41791 | Kochi | IND | 0.0000055 | 12.64179 | Sikka | IND | 0 | 0 |
| Haifa | ISR | 0.0000530 | 121.82090 | Sokhna | EGY | 0.0000050 | 11.49254 | Onne | NGA | 0 | 0 |
| El Dekheila | EGY | 0.0000515 | 118.37313 | Kandla | IND | 0.0000040 | 9.19403 | Dakar | SEN | 0 | 0 |
| Port Sudan | SDN | 0.0000490 | 112.62687 | Tarragona | ESP | 0.0000040 | 9.19403 | Casablanca | MAR | 0 | 0 |
| Ashdod | ISR | 0.0000480 | 110.32836 | Kakinada | IND | 0.0000040 | 9.19403 | Motril | ESP | 0 | 0 |
| Bilbao | ESP | 0.0000445 | 102.28358 | Jubail | SAU | 0.0000020 | 4.59701 | Seville | ESP | 0 | 0 |

***** denotes the relative pest’s arrival rate versus the avergae *ϕ_ij_* values for all network locations (i.e. the mean of all *ϕ_ij_* values in Tables S3-S12) ($\overline{\phi}_{ij}$ = 0.00259)
