# Supplementary material for: Modelling the Arrival of Invasive Organisms via the International Marine Shipping Network: A Khapra Beetle Study
Source: PLoS One. 2012 Sep 6;7(9):e44589. doi: 10.1371/journal.pone.0044589 (PMC3435288; doi:10.1371/journal.pone.0044589)
Supplement: Table S10 — Ranking of all source ports for Khapra beetle introduction to the Australian port of Sydney Harbour. (DOCX) [file pone.0044589.s010.docx]

Table S10. Ranking of all source ports for Khapra beetle introduction to the Australian port of Sydney Harbour.

| **Sydney Harbour** |  |  |  |  |  |  |  |  |  |  |  |
| --- | --- | --- | --- | --- | --- | --- | --- | --- | --- | --- | --- |
| **Port of origin *i*** | **Country** | ***ϕ_ij_*** | **relative *ϕ_ij_**** | **Port of origin *i*** | **Country** | ***ϕ_ij_*** | **relative *ϕ_ij_**** | **Port of origin *i*** | **Country** | ***ϕ_ij_*** | **relative *ϕ_ij_**** |
| Busan | KOR | 0.0084035 | 19315.50746 | Port Muhammad Bin Qasim | PAK | 0.0000280 | 64.35821 | Mai-Liao | TWN | 0.0000010 | 2.29851 |
| Kaohsiung | TWN | 0.0076975 | 17692.76119 | Bilbao | ESP | 0.0000280 | 64.35821 | Haldia | IND | 0.0000005 | 1.14925 |
| Keelung | TWN | 0.0025470 | 5854.29851 | Ambarli | TUR | 0.0000275 | 63.20896 | Pasajes | ESP | 0.0000005 | 1.14925 |
| Damietta | EGY | 0.0017950 | 4125.82090 | Hodeidah | YEM | 0.0000260 | 59.76119 | Jubail | SAU | 0.0000005 | 1.14925 |
| Colombo | LKA | 0.0008910 | 2047.97015 | Limassol | CYP | 0.0000245 | 56.31343 | Sokhna | EGY | 0.0000005 | 1.14925 |
| Jeddah | SAU | 0.0006950 | 1597.46269 | Haifa | ISR | 0.0000240 | 55.16418 | Mongla | BGD | 0.0000005 | 1.14925 |
| Valencia | ESP | 0.0006575 | 1511.26866 | Yarimca | TUR | 0.0000210 | 48.26866 | Nouakchott | MRT | 0.0000005 | 1.14925 |
| Port Said | EGY | 0.0004950 | 1137.76119 | Istanbul | TUR | 0.0000200 | 45.97015 | Yanbu | SAU | 0 | 0 |
| Ulsan | KOR | 0.0003795 | 872.28358 | New Tuticorin | IND | 0.0000185 | 42.52239 | Malaga | ESP | 0 | 0 |
| Barcelona | ESP | 0.0002920 | 671.16418 | Izmir | TUR | 0.0000150 | 34.47761 | Bandirma | TUR | 0 | 0 |
| Gwangyang | KOR | 0.0002500 | 574.62687 | Montevideo | URY | 0.0000140 | 32.17910 | Mukalla | YEM | 0 | 0 |
| Algeciras | ESP | 0.0001930 | 443.61194 | Gemlik | TUR | 0.0000130 | 29.88060 | Eilat | ISR | 0 | 0 |
| Jawaharlal Nehru | IND | 0.0001860 | 427.52239 | Suez | EGY | 0.0000125 | 28.73134 | Mundra | IND | 0 | 0 |
| Apapa-Lagos | NGA | 0.0001330 | 305.70149 | Yosu | KOR | 0.0000115 | 26.43284 | Algiers | DZA | 0 | 0 |
| Aden | YEM | 0.0001150 | 264.32836 | Ashkelon | ISR | 0.0000110 | 25.28358 | Samho | KOR | 0 | 0 |
| Karachi | PAK | 0.0000940 | 216.05970 | Alexandria | EGY | 0.0000110 | 25.28358 | Santander | ESP | 0 | 0 |
| Dammam | SAU | 0.0000830 | 190.77612 | Cadiz | ESP | 0.0000090 | 20.68657 | Ceuta | ESP | 0 | 0 |
| Taichung | TWN | 0.0000800 | 183.88060 | Beirut | LBN | 0.0000080 | 18.38806 | Ras Lanuf | LBY | 0 | 0 |
| Mumbai | IND | 0.0000785 | 180.43284 | Chittagong | BGD | 0.0000075 | 17.23881 | Pyeongtaek | KOR | 0 | 0 |
| Masan | KOR | 0.0000680 | 156.29851 | Kolkata | IND | 0.0000065 | 14.94030 | Donghae | KOR | 0 | 0 |
| Chennai | IND | 0.0000670 | 154.00000 | Kandla | IND | 0.0000060 | 13.79104 | Lattakia | SYR | 0 | 0 |
| Port Sudan | SDN | 0.0000535 | 122.97015 | Kochi | IND | 0.0000055 | 12.64179 | Alang | IND | 0 | 0 |
| Bandar Abbas | IRN | 0.0000520 | 119.52239 | Visakhapatnam | IND | 0.0000050 | 11.49254 | Karwar | IND | 0 | 0 |
| Incheon | KOR | 0.0000405 | 93.08955 | Palma | ESP | 0.0000035 | 8.04478 | Sikka | IND | 0 | 0 |
| Mersin | TUR | 0.0000395 | 90.79104 | Tripoli | LBY | 0.0000020 | 4.59701 | Onne | NGA | 0 | 0 |
| Arzew | DZA | 0.0000345 | 79.29851 | Derince | TUR | 0.0000015 | 3.44776 | Dakar | SEN | 0 | 0 |
| El Dekheila | EGY | 0.0000330 | 75.85075 | Ain Sukhna Term. | EGY | 0.0000015 | 3.44776 | Casablanca | MAR | 0 | 0 |
| Ashdod | ISR | 0.0000320 | 73.55224 | Tuzla | TUR | 0.0000015 | 3.44776 | Motril | ESP | 0 | 0 |
| Tarragona | ESP | 0.0000320 | 73.55224 | Kakinada | IND | 0.0000010 | 2.29851 | Seville | ESP | 0 | 0 |

***** denotes the relative pest’s arrival rate versus the avergae *ϕ_ij_* values for all network locations (i.e. the mean of all *ϕ_ij_* values in Tables S3-S12) ($\overline{\phi}_{ij}$ = 0.00259)
