# Supplementary material for: Modelling the Arrival of Invasive Organisms via the International Marine Shipping Network: A Khapra Beetle Study
Source: PLoS One. 2012 Sep 6;7(9):e44589. doi: 10.1371/journal.pone.0044589 (PMC3435288; doi:10.1371/journal.pone.0044589)
Supplement: Table S11 — Ranking of all source ports for Khapra beetle introduction to the Australian port of Hobart. (DOCX) [file pone.0044589.s011.docx]

Table S11. Ranking of all source ports for Khapra beetle introduction to the Australian port of Hobart.

| **Hobart** |  |  |  |  |  |  |  |  |  |  |  |
| --- | --- | --- | --- | --- | --- | --- | --- | --- | --- | --- | --- |
| **Port of origin *i*** | **Country** | ***ϕ_ij_*** | **relative *ϕ_ij_**** | **Port of origin *i*** | **Country** | ***ϕ_ij_*** | **relative *ϕ_ij_**** | **Port of origin *i*** | **Country** | ***ϕ_ij_*** | **relative *ϕ_ij_**** |
| Busan | KOR | 0.0048670 | 11186.83582 | Ashdod | ISR | 0.0000120 | 27.58209 | Mongla | BGD | 0.0000005 | 1.14925 |
| Kaohsiung | TWN | 0.0041040 | 9433.07463 | Bilbao | ESP | 0.0000110 | 25.28358 | Kakinada | IND | 0 | 0 |
| Keelung | TWN | 0.0014075 | 3235.14925 | Ambarli | TUR | 0.0000105 | 24.13433 | Pasajes | ESP | 0 | 0 |
| Damietta | EGY | 0.0006960 | 1599.76119 | Istanbul | TUR | 0.0000105 | 24.13433 | Jubail | SAU | 0 | 0 |
| Ulsan | KOR | 0.0005480 | 1259.58209 | Port Muhammad Bin Qasim | PAK | 0.0000100 | 22.98507 | Mai-Liao | TWN | 0 | 0 |
| Colombo | LKA | 0.0003945 | 906.76119 | Montevideo | URY | 0.0000090 | 20.68657 | Malaga | ESP | 0 | 0 |
| Valencia | ESP | 0.0003520 | 809.07463 | Izmir | TUR | 0.0000085 | 19.53731 | Sokhna | EGY | 0 | 0 |
| Jeddah | SAU | 0.0003500 | 804.47761 | Suez | EGY | 0.0000080 | 18.38806 | Bandirma | TUR | 0 | 0 |
| Port Said | EGY | 0.0002160 | 496.47761 | Yosu | KOR | 0.0000080 | 18.38806 | Tuzla | TUR | 0 | 0 |
| Barcelona | ESP | 0.0001265 | 290.76119 | Gemlik | TUR | 0.0000075 | 17.23881 | Mukalla | YEM | 0 | 0 |
| Gwangyang | KOR | 0.0001115 | 256.28358 | Beirut | LBN | 0.0000075 | 17.23881 | Nouakchott | MRT | 0 | 0 |
| Algeciras | ESP | 0.0000730 | 167.79104 | New Tuticorin | IND | 0.0000065 | 14.94030 | Eilat | ISR | 0 | 0 |
| Jawaharlal Nehru | IND | 0.0000565 | 129.86567 | Ashkelon | ISR | 0.0000060 | 13.79104 | Mundra | IND | 0 | 0 |
| Aden | YEM | 0.0000500 | 114.92537 | Alexandria | EGY | 0.0000060 | 13.79104 | Algiers | DZA | 0 | 0 |
| Dammam | SAU | 0.0000400 | 91.94030 | Yarimca | TUR | 0.0000045 | 10.34328 | Samho | KOR | 0 | 0 |
| Taichung | TWN | 0.0000400 | 91.94030 | Cadiz | ESP | 0.0000045 | 10.34328 | Santander | ESP | 0 | 0 |
| Karachi | PAK | 0.0000365 | 83.89552 | Chittagong | BGD | 0.0000035 | 8.04478 | Ceuta | ESP | 0 | 0 |
| Chennai | IND | 0.0000355 | 81.59701 | Visakhapatnam | IND | 0.0000025 | 5.74627 | Ras Lanuf | LBY | 0 | 0 |
| Masan | KOR | 0.0000340 | 78.14925 | Kolkata | IND | 0.0000025 | 5.74627 | Pyeongtaek | KOR | 0 | 0 |
| Bandar Abbas | IRN | 0.0000295 | 67.80597 | Palma | ESP | 0.0000020 | 4.59701 | Donghae | KOR | 0 | 0 |
| Incheon | KOR | 0.0000280 | 64.35821 | Tripoli | LBY | 0.0000020 | 4.59701 | Lattakia | SYR | 0 | 0 |
| Apapa-Lagos | NGA | 0.0000225 | 51.71642 | Kochi | IND | 0.0000015 | 3.44776 | Alang | IND | 0 | 0 |
| Haifa | ISR | 0.0000165 | 37.92537 | Tarragona | ESP | 0.0000015 | 3.44776 | Karwar | IND | 0 | 0 |
| Mersin | TUR | 0.0000160 | 36.77612 | Haldia | IND | 0.0000015 | 3.44776 | Sikka | IND | 0 | 0 |
| Limassol | CYP | 0.0000135 | 31.02985 | Derince | TUR | 0.0000010 | 2.29851 | Onne | NGA | 0 | 0 |
| Mumbai | IND | 0.0000135 | 31.02985 | Arzew | DZA | 0.0000010 | 2.29851 | Dakar | SEN | 0 | 0 |
| Port Sudan | SDN | 0.0000125 | 28.73134 | Kandla | IND | 0.0000005 | 1.14925 | Casablanca | MAR | 0 | 0 |
| El Dekheila | EGY | 0.0000125 | 28.73134 | Ain Sukhna Term. | EGY | 0.0000005 | 1.14925 | Motril | ESP | 0 | 0 |
| Hodeidah | YEM | 0.0000125 | 28.73134 | Yanbu | SAU | 0.0000005 | 1.14925 | Seville | ESP | 0 | 0 |

***** denotes the relative pest’s arrival rate versus the avergae *ϕ_ij_* values for all network locations (i.e. the mean of all *ϕ_ij_* values in Tables S3-S12) ($\overline{\phi}_{ij}$ = 0.00259)
