# Supplementary material for: Modelling the Arrival of Invasive Organisms via the International Marine Shipping Network: A Khapra Beetle Study
Source: PLoS One. 2012 Sep 6;7(9):e44589. doi: 10.1371/journal.pone.0044589 (PMC3435288; doi:10.1371/journal.pone.0044589)
Supplement: Table S12 — Ranking of all source ports for Khapra beetle introduction to the Australian port of Newcastle. (DOCX) [file pone.0044589.s012.docx]

Table S12. Ranking of all source ports for Khapra beetle introduction to the Australian port of Newcastle.

| **Newcastle** |  |  |  |  |  |  |  |  |  |  |  |
| --- | --- | --- | --- | --- | --- | --- | --- | --- | --- | --- | --- |
| **Port of origin *i*** | **Country** | ***ϕ_ij_*** | **relative *ϕ_ij_**** | **Port of origin *i*** | **Country** | ***ϕ_ij_*** | **relative *ϕ_ij_**** | **Port of origin *i*** | **Country** | ***ϕ_ij_*** | **relative *ϕ_ij_**** |
| Busan | KOR | 0.0008935 | 2053.71642 | Yarimca | TUR | 0.0000045 | 10.34328 | Suez | EGY | 0 | 0 |
| Kaohsiung | TWN | 0.0008225 | 1890.52239 | Port Sudan | SDN | 0.0000030 | 6.89552 | Derince | TUR | 0 | 0 |
| Keelung | TWN | 0.0002775 | 637.83582 | Port Muhammad Bin Qasim | PAK | 0.0000030 | 6.89552 | Tarragona | ESP | 0 | 0 |
| Damietta | EGY | 0.0001785 | 410.28358 | El Dekheila | EGY | 0.0000025 | 5.74627 | Ain Sukhna Term. | EGY | 0 | 0 |
| Colombo | LKA | 0.0000935 | 214.91045 | Hodeidah | YEM | 0.0000025 | 5.74627 | Pasajes | ESP | 0 | 0 |
| Valencia | ESP | 0.0000865 | 198.82090 | Ashdod | ISR | 0.0000025 | 5.74627 | Jubail | SAU | 0 | 0 |
| Jeddah | SAU | 0.0000665 | 152.85075 | Limassol | CYP | 0.0000025 | 5.74627 | Yanbu | SAU | 0 | 0 |
| Barcelona | ESP | 0.0000505 | 116.07463 | Mumbai | IND | 0.0000020 | 4.59701 | Malaga | ESP | 0 | 0 |
| Port Said | EGY | 0.0000390 | 89.64179 | New Tuticorin | IND | 0.0000020 | 4.59701 | Mukalla | YEM | 0 | 0 |
| Karachi | PAK | 0.0000365 | 83.89552 | Ashkelon | ISR | 0.0000020 | 4.59701 | Mongla | BGD | 0 | 0 |
| Ulsan | KOR | 0.0000345 | 79.29851 | Beirut | LBN | 0.0000020 | 4.59701 | Nouakchott | MRT | 0 | 0 |
| Arzew | DZA | 0.0000340 | 78.14925 | Montevideo | URY | 0.0000015 | 3.44776 | Eilat | ISR | 0 | 0 |
| Ambarli | TUR | 0.0000330 | 75.85075 | Bilbao | ESP | 0.0000010 | 2.29851 | Mundra | IND | 0 | 0 |
| Bandirma | TUR | 0.0000320 | 73.55224 | Alexandria | EGY | 0.0000010 | 2.29851 | Algiers | DZA | 0 | 0 |
| Chittagong | BGD | 0.0000280 | 64.35821 | Cadiz | ESP | 0.0000010 | 2.29851 | Samho | KOR | 0 | 0 |
| Kakinada | IND | 0.0000260 | 59.76119 | Visakhapatnam | IND | 0.0000010 | 2.29851 | Santander | ESP | 0 | 0 |
| Gwangyang | KOR | 0.0000240 | 55.16418 | Kolkata | IND | 0.0000010 | 2.29851 | Ceuta | ESP | 0 | 0 |
| Jawaharlal Nehru | IND | 0.0000185 | 42.52239 | Kochi | IND | 0.0000010 | 2.29851 | Ras Lanuf | LBY | 0 | 0 |
| Algeciras | ESP | 0.0000180 | 41.37313 | Tripoli | LBY | 0.0000010 | 2.29851 | Pyeongtaek | KOR | 0 | 0 |
| Aden | YEM | 0.0000165 | 37.92537 | Istanbul | TUR | 0.0000005 | 1.14925 | Donghae | KOR | 0 | 0 |
| Taichung | TWN | 0.0000105 | 24.13433 | Gemlik | TUR | 0.0000005 | 1.14925 | Lattakia | SYR | 0 | 0 |
| Chennai | IND | 0.0000105 | 24.13433 | Yosu | KOR | 0.0000005 | 1.14925 | Alang | IND | 0 | 0 |
| Dammam | SAU | 0.0000095 | 21.83582 | Kandla | IND | 0.0000005 | 1.14925 | Karwar | IND | 0 | 0 |
| Masan | KOR | 0.0000070 | 16.08955 | Palma | ESP | 0.0000005 | 1.14925 | Sikka | IND | 0 | 0 |
| Haifa | ISR | 0.0000055 | 12.64179 | Haldia | IND | 0.0000005 | 1.14925 | Onne | NGA | 0 | 0 |
| Bandar Abbas | IRN | 0.0000050 | 11.49254 | Mai-Liao | TWN | 0.0000005 | 1.14925 | Dakar | SEN | 0 | 0 |
| Incheon | KOR | 0.0000050 | 11.49254 | Sokhna | EGY | 0.0000005 | 1.14925 | Casablanca | MAR | 0 | 0 |
| Mersin | TUR | 0.0000045 | 10.34328 | Tuzla | TUR | 0.0000005 | 1.14925 | Motril | ESP | 0 | 0 |
| Apapa-Lagos | NGA | 0.0000045 | 10.34328 | Izmir | TUR | 0 | 0 | Seville | ESP | 0 | 0 |

***** denotes the relative pest’s arrival rate versus the avergae *ϕ_ij_* values for all network locations (i.e. the mean of all *ϕ_ij_* values in Tables S3-S12) ($\overline{\phi}_{ij}$= 0.00259)
